# Targeting regulated cell death (RCD) with small-molecule compounds in triple-negative breast cancer: a revisited perspective from molecular mechanisms to targeted therapies

**DOI:** 10.1186/s13045-022-01260-0

**Published:** 2022-04-12

**Authors:** Minru Liao, Rui Qin, Wei Huang, Hong-Ping Zhu, Fu Peng, Bo Han, Bo Liu

**Affiliations:** 1 State Key Laboratory of Biotherapy and Cancer Center, West China Hospital, and West China School of Pharmacy, Sichuan University, Chengdu, 610041 China; 2 State Key Laboratory of Southwestern Chinese Medicine Resources, Hospital of Chengdu University of Traditional Chinese Medicine, School of Pharmacy, Chengdu University of Traditional Chinese Medicine, Chengdu, 611137 China; 3 Antibiotics Research and Re-Evaluation Key Laboratory of Sichuan Province, Sichuan Industrial Institute of Antibiotics, Chengdu University, Chengdu, China

**Keywords:** Triple-negative breast cancer (TNBC), Regulated cell death (RCD), Autophagy-dependent cell death, Apoptosis, Necroptosis, Ferroptosis, Mitotic catastrophe, Pyroptosis, Anoikis, Combination strategy

## Abstract

Triple-negative breast cancer (TNBC) is a subtype of human breast cancer with one of the worst prognoses, with no targeted therapeutic strategies currently available. Regulated cell death (RCD), also known as programmed cell death (PCD), has been widely reported to have numerous links to the progression and therapy of many types of human cancer. Of note, RCD can be divided into numerous different subroutines, including autophagy-dependent cell death, apoptosis, mitotic catastrophe, necroptosis, ferroptosis, pyroptosis and anoikis. More recently, targeting the subroutines of RCD with small-molecule compounds has been emerging as a promising therapeutic strategy, which has rapidly progressed in the treatment of TNBC. Therefore, in this review, we focus on summarizing the molecular mechanisms of the above-mentioned seven major RCD subroutines related to TNBC and the latest progress of small-molecule compounds targeting different RCD subroutines. Moreover, we further discuss the combined strategies of one drug (e.g., narciclasine) or more drugs (e.g., torin-1 combined with chloroquine) to achieve the therapeutic potential on TNBC by regulating RCD subroutines. More importantly, we demonstrate several small-molecule compounds (e.g., ONC201 and NCT03733119) by targeting the subroutines of RCD in TNBC clinical trials. Taken together, these findings will provide a clue on illuminating more actionable low-hanging-fruit druggable targets and candidate small-molecule drugs for potential RCD-related TNBC therapies.

**Graphical abstract:**

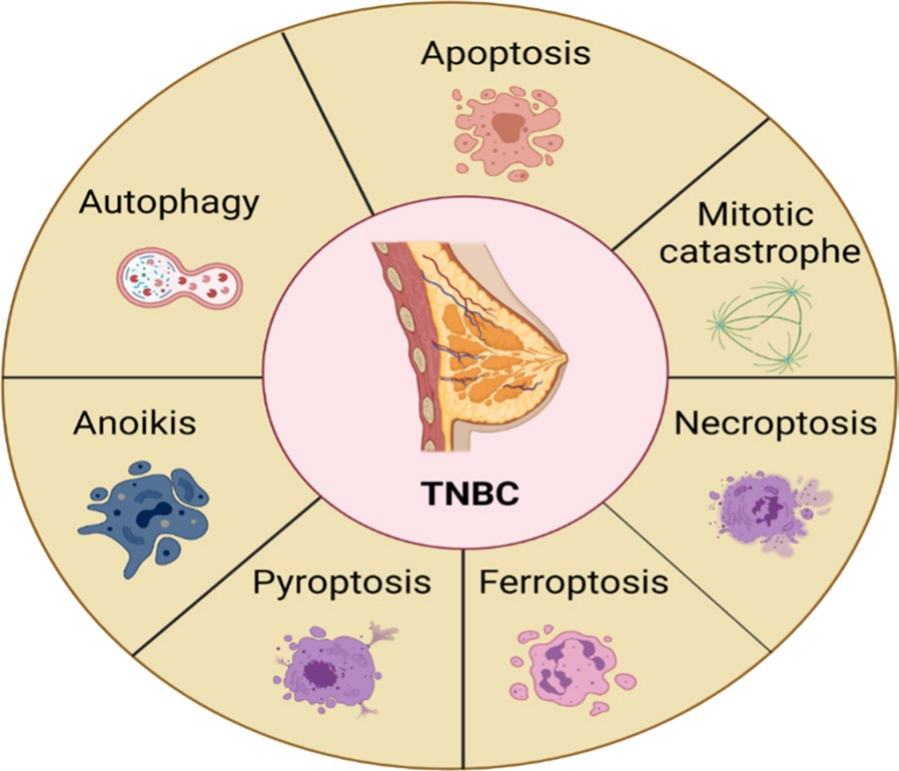

## Introduction

Hitherto, breast cancer has attained the highest cancer incidence in the world and is also the leading cause of cancer-related death among women worldwide. Based upon gene expression profiles, breast cancer can be divided into five clinical types. According to the status of estrogen receptor (ER), progesterone receptor (PR), and human epidermal growth factor 2 (HER2), the subtypes can be defined as normal-like breast cancer, luminal A breast cancer, luminal B breast cancer, HER2-enriched breast cancer and triple-negative/basal-like breast cancer [1]. Triple-negative breast cancer (TNBC) is the breast cancer subtype with the worst prognosis, and it has a strong invasive and metastatic capacity and easily invaded into blood vessels [2], increasing the recurrence rate. Due to the lack of expressions of ER, PR and HER2, endocrine and targeted therapies achieve comparatively poor outcomes. Therapeutic methods for TNBC are much more limited compared with other breast cancers. Based upon the gene expression profiles of TNBC cases in breast cancer datasets, seven TNBC subtypes were identified: basal-like 1 (BL1), basal-like 2 (BL2), immunomodulatory (IM), mesenchymal (M), mesenchymal stem-like, luminal androgen receptor, and unclassified (UNS) [3]. The tumors of patients with TNBC show a high genetic diversity ranging from highly proliferative tumors to chemotherapy-resistant tumors with low proliferation and luminal characteristics [4]. These data may be useful for biomarker selection, drug discovery and clinical trial design [5], with the aim of matching patients with TNBC of different subtypes with the appropriate targeted therapies.

In the past decade, the Nomenclature of Cell Death Committee (NCCD) has developed the guidelines for defining and interpreting cell death from the perspectives of morphology, biochemistry and function [6–8]. The NCCD divides cell death into accidental death (ACD) and regulated cell death (RCD) [8]. ACD is the instantaneous and catastrophic death of cells under the natural conditions, such as physical stressors (e.g., high pressure, temperature or osmotic force), chemical stressors (e.g., extreme pH change) and mechanical stressors (e.g., shear force). RCD depends on specific molecular mechanisms and is subject to regulation. RCD is also called programmed cell death (PCD) during development or tissue renewal [8], as it plays a key role in these processes. RCD is crucial in the response to injury, infection and inflammation, in this case involving an intracellular suicide pathway [9]. According to its different mechanisms [10], RCD can be divided into three major categories: autophagy, apoptosis and other types of RCD involved in signaling pathways, such as mitotic catastrophe, necroptosis, ferroptosis, pyroptosis and anoikis [11]. RCD can be regulated by pharmacological agents and genetic programs [12] and plays a vital role in tissue homeostasis. Aberrant regulation of this process can be related to a variety of diseases, especially cancer [13, 14].

TNBC has given a rise to the largest proportion in breast cancer-related death, exerting higher recurrence, more aggressive growth and more rapid metastasis. Since TNBC is the absence of hormone receptor and HER2, TNBC patients cannot respond to hormone therapy or any other available targeted agents; therefore, it is imperative to search for innovative therapeutic targets for TNBC. In recent years, a series of small-molecule compounds targeting RCD have made a good progress in the clinical treatment of TNBC. In this review, we summarized the molecular mechanisms of seven major RCD subroutines related to TNBC and the latest progress of small-molecule compounds targeting different RCD subroutines. We also discussed the combined strategies of one drug or more drugs by regulating RCD subroutines in TNBC therapy. Moreover, we demonstrated small-molecule compounds by targeting the subroutines of RCD in TNBC clinical trials.

## Targeting apoptotic pathways with small-molecule compounds in TNBC

**Fig. 1:**
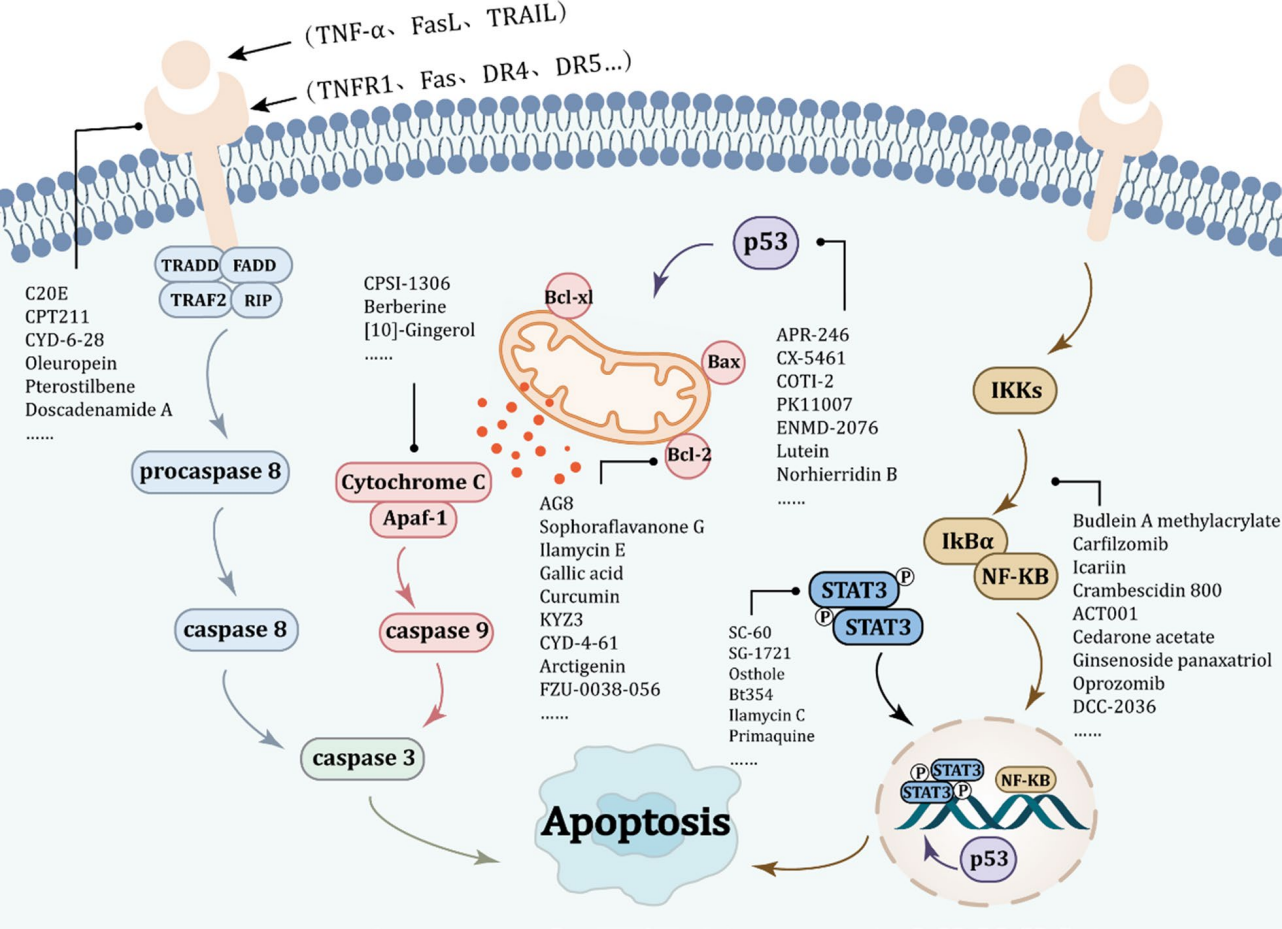
Core apoptotic signaling pathways in triple negative breast cancer (TNBC). In the extrinsic pathway, the interaction between death receptors and their ligands activates caspase 8, which then activates caspase 3, and eventually lead to apoptosis. Death receptors belong to tumor necrosis factor (TNF) receptors superfamily, which is essential for the transmission of intracellular and extracellular signals. The death receptors include FAS, TNFR1, DR4, and DR5. Death receptors bind to the corresponding proapoptotic ligand like FASL, TNF-α, and TNF-related apoptosis-inducing ligand (TRAIL), to trigger extrinsic apoptosis. In mitochondrial dependent apoptotic pathway, when DNA damage occurs, the pro-apoptotic proteins of the Bcl-2 family (such as Bax and Bak) will be upregulated and activated. Anti-apoptotic proteins (such as Bcl-2 and Bcl-xl) inhibits the action of Bax and Bak. A series of apoptogenic factors will be released into the cytosol, including cytochrome c, apaf-1, and procaspase 9, which forms a complex called apoptosome. This complex can activate caspase 9 followed by the transformation of pro-caspase 3 to caspase 3 and thus trigger apoptosis. When cells receive extracellular stimulation, they transmit the signal to inhibitor of kappa-B kinase (IKK), and inhibitor of IκB is separated from the trimer complex formed with NF-κB. The released NF-κB rapidly enters the nucleus and binds to specific sequences on deoxyribonucleic acid (DNA) to participate in physiological processes such as anti-apoptotic effects. Besides, apoptosis can also be induced by regulating the expression of p53 protein. Abbreviations: Apaf-1: Apoptotic protease activating factor 1; Bcl-2: B-cell lymphoma 2; Bcl-xl: B-cell lymphoma-extra large; DR4/5: Death receptor 4/5; FADD: Fas/fas associated via death domain; IκBα: Nuclear factor kappa-B inhibitor α; NF-κB: Nuclear factor kappa-B; RIP: Receptor-interacting protein; STAT3: Signal transducer and activator of transcription 3; TNF-α: Tumor necrosis factor-α; TNFR1: tumor necrosis factor receptor1; TRADD: TNFRSF1A associated via death domain; TRAF2: TNF receptor-associated factor 2; TRAIL: TNF-related apoptosis-inducing ligand

Apoptosis is the main type of programmed cell death (PCD) and has increasingly becoming an essential target for antitumor drug screening [15]. Apoptosis of cancer cells is mainly activated by the following two pathways: extrinsic pathway and intrinsic pathway [16]. The extrinsic pathway is initiated by members of the death receptor family. The binding between death receptors and their ligands leads to the formation of the death-inducing signaling complex (DISC), which eventually activates caspase-8 [17]. The intrinsic (also called mitochondrial) pathway is activated by deoxyribonucleic acid (DNA) damage, growth factor withdrawal, change of osmotic pressure and cytotoxic stimulation of anticancer agents and then regulates the expression of pro-apoptotic and anti-apoptotic proteins, thereby activating caspase-9 and caspase-3 (Fig. 1) [18, 19].

In the extrinsic pathway, the interaction between death receptors and their ligands activates caspase-8, which then activates caspase-3 and eventually leads to apoptosis. Death receptors belong to tumor necrosis factor (TNF) receptors superfamily, which is essential for the transmission of intracellular and extracellular signals. The death receptors include FAS, TNFR1, DR4 and DR5. Death receptors bind to the corresponding proapoptotic ligand like FASL, TNF-α and TNF-related apoptosis-inducing ligand (TRAIL), to trigger extrinsic apoptosis. In mitochondrial-dependent apoptotic pathway, when DNA damage occurs, the pro-apoptotic proteins of the Bcl-2 family (such as Bax and Bak) will be upregulated and activated. Anti-apoptotic proteins (such as Bcl-2 and Bcl-xl) inhibit the action of Bax and Bak. A series of apoptogenic factors will be released into the cytosol, including cytochrome c, apaf-1 and procaspase 9, which forms a complex called apoptosome. This complex can activate caspase-9 followed by the transformation of pro-caspase-3 to caspase-3 and thus trigger apoptosis. When cells receive extracellular stimulation, they transmit the signal to inhibitor of kappa-B kinase (IKK), and inhibitor of IκB is separated from the trimer complex formed with NF-κB. The released NF-κB rapidly enters the nucleus and binds to specific sequences on deoxyribonucleic acid (DNA) to participate in physiological processes such as anti-apoptotic effects. Besides, apoptosis can also be induced by regulating the expression of p53 protein.

Small-molecule drugs play an important role in the treatment of cancer, which combine with specific target molecules in cells to exert their specific functions, such as inducing apoptosis of cancer cells [20, 21]. Targeting the key regulators of apoptosis with the goal of inducing apoptosis in cancer cells was one of the most attractive strategies for drug discovery and development, as well as the hot area of oncology research [22]. Small-molecule drugs had been the focus of research due to their strong specificity, remarkable effect and less damage to normal cells [23, 24]. Small-molecule compounds summarized in this paper include not only drugs that have entered clinical trials, but also synthetic compounds, natural compounds and semisynthetic derivatives of natural compounds. It was estimated that about 60% of marketed drugs were natural compounds or semisynthetic derivatives of natural compounds [25, 26]. Next, we will describe the antitumor activity of small-molecule compounds in TNBC in terms of apoptosis-related signaling pathways and targets, and the main pathways and targets of apoptosis include tumor necrosis factor receptors (TNFR), B-cell lymphoma 2 (Bcl-2) family, apoptotic protease activating factor 1 (Apaf-1) and cytochrome c (Cyt-C), nuclear factor kappa-B (NF-κB) pathway, signal transducer and activator of transcription-3 (STAT3) pathway and p53.

### Targeting TNF-related ligands and their receptors

The TNFR family has many members, including Fas, TNFR1, death receptor (DR) 4, and DR5, which bound to their ligands FasL, TNF-α and TNF-related apoptosis-inducing ligand (TRAIL), respectively, triggering a signaling cascade that resulted in the recruitment and activation of caspase-8, ultimately inducing cell apoptosis and causing programmed cell death [27–29]. The TNFR had been studied for many years as a potential target for tumor therapy, among which TNF-α and TRAIL had been the most studied [30]. Recently, investigations gradually identified the regulatory effect of small-molecule compounds on TNF-related receptors and their ligands, leading to the induction of apoptosis in TNBC.

V-3–17,18-epoxyeicosatetraenoic acid (C20E), a newly synthesized compound, could stimulate TNFR-1/ASK1/JNK signaling to induce apoptosis of MDA-MB-231 cells, which acted at the intracellular domain of TNFR1, and then activated TNFRSF1A associated via death domain (TRADD), TNF receptor-associated factor 2 (TRAF2) and several downstream signal. Besides, the anti-breast cancer activity of C20E in vivo also relied on its modulatory effect on TNFR-1/ASK1/JNK signaling and apoptosis induction [31]. In addition, CPT211, a novel camptothecin derivative, was found to suppress the proliferation and induce apoptosis of MDA-MB-231 cells effectively, by activating Fas/fas associated via death domain (FADD)/caspase-8 signaling [32].

TRAIL, a proapoptotic molecule, can selectively induce apoptosis in a variety of human tumor cell lines without affecting normal cells [33]. Most small-molecule compounds could be used in combination with TRAIL to promote apoptosis of TNBC cells. For example, pterostilbene (PTER), a natural analogue of resveratrol, was proved to enhance TRAIL-induced apoptosis via reactive oxygen species (ROS)-mediated C/EBP homologous protein (CHOP) activation, leading to the expression of DR4 and DR5 [34]. Like PTER, doscadenamide A could also synergistically interact with TRAIL that induced exogenous apoptosis, and bound to death receptors to induce apoptosis of TNBC cells [35].

Organometallic complexes exhibit a significant anti-breast cancer activity and induced apoptosis against TNBC cells [36]. For an instance, Mn^III^ complex could enhance the activity of caspase-8 and caspase-9, upregulate the expression of Bax/Bcl-2 ratio and promote the binding of TNF-α to its receptor, indicating a simultaneous activation of both internal and external apoptotic pathways in MDA-MB-231 cells. In addition, Mn^III^ complex combined with Adriamycin could synergistically inhibit the growth of TNBC cells [37]. Likewise, numerous small-molecule compounds could trigger apoptosis of TNBC cell lines by influencing TNF-α-related pathway [38, 39] (Table 1).

**Table 1:** Small-molecule compounds targeting apoptosis in TNBC

Name in the literature and chemical structure	Target	Mechanism in RCD	Biological activity	TNBC subtype	References
C20E	TNFR1/ASK1/JNK↑	Induce apoptosis	MDA-MB-231 (IC_50_ = 40 μM)	MSL	[31]
CPT211	Fas/FADD/caspase-8↑	Induce apoptosis	MDA-MB-231 (IC_50_ = 478.4 nM)	MSL	[32]
Pterostilbene (PTER)	DR4/DR5↑ Cellular FLICE-inhibitory protein (c-FLIP)↓ Bcl-xl↓ Bcl-2↓	Induce apoptosis	MDA-MB-468 (IC_50_ = 30 − 40 μM); BT-20 (IC_50_ = 50 − 60 μM)	BL1	[34]
Doscadenamide A	TRAIL/DR↑	Induce apoptosis	MDA-MB-231	MSL	[35]
Mn^III^ complex	TNF-α/TNFR↑ Bcl-2↓ Bax↑ caspase-8,9↑	Induce apoptosis	MDA-MB-231 (IC_50_ = 2.28 ± 0.38 μM)	MSL	[37]
CYD-6–28	DR5↑	Induce apoptosis	HCC1806, HCC1937 (IC_50_ = 2 − 3 μM)	BL2, BL1	[38]
MEDI3039	DR5↑	Induce apoptosis	MDA-MB-231 (IC_50_ = 4.71 μM)	MSL	[39]
Spirooxindole 6e	Bcl-2↓ Bax↑ caspase-3↑	Induce apoptosis	MDA-MB-231 (IC_50_ = 6.40 μM)	MSL	[46]
Compound 10 g	Bcl-2↓ Bax↑ caspase-3,9↑	Induce apoptosis	MDA-MB-231 (IC_50_ = 12.0 ± 0.13 μM)	MSL	[47]
Sophoraflavanone G (SG)	Bcl-2↓ Bcl-xl↓ ERK/AKT↓ Bax↑ caspase-3↑ Cyt-c↑	Induce apoptosis	MDA-MB-231 (IC_50_ = 29.7 ± 5.2 μM)	MSL	[48]
Ilamycin E	CHOP↑ Bcl-2↓	Induce apoptosis	HCC1806 (IC_50_ = 47.50 μM), HCC1937 (IC_50_ = 14.24 μM), MDA-MB-468 (IC_50_ = 24.56 μM), MDA-MB-231 (IC_50_ = 33.72 μM)	BL2, BL1, MSL	[49]
Gallic acid (GA)	Bax↑ caspase-3↑ Bcl-2↓	Induce apoptosis	MDA-MB-231 (IC_50_ = 50 μM)	MSL	[50]
Curcumin (Cur)	Bax↑ caspase-3↑ Bcl-2↓	Induce apoptosis	MDA-MB-231 (IC_50_ = 30 μM)	MSL	[50]
KYZ3	Bcl-2↓ Bax↑	Induce apoptosis	MDA-MB-231 (IC_50_ = 0.68 μM), MDA-MB-468 (IC_50_ = 0.86 μM)	MSL, BL1	[51]
Compound 7d	Bax↑ Bim↑ Cyt-c↑	Induce apoptosis	MDA-MB-231 (IC_50_ = 0.25 μM), SUM159 (IC_50_ = 0.20 μM), 4T1 (IC_50_ = 0.22 μM)	MSL	[52]
CYD-4–61	Bax↑ Cyt-c↑	Induce apoptosis	MDA-MB-231 (IC_50_ = 0.07 μM), MDA-MB-468 (IC_50_ = 1.61 μM)	MSL, BL1	[53]
Arctigenin (ATG) (+ DOX)	Bcl-2↓ Bcl-xl↓ Bax↑	Induce apoptosis	MDA-MB-231 (ATG: IC_50_ = 10 − 25 μM; DOX: IC_50_ = 0.2 μM)	MSL	[58]
Coralyne (+ PTX)	Bcl-2↓ Bax↑	Induce apoptosis	MDA-MB-231 (Coralyne: IC_50_ = 21.9 ± 1.36 μM; PTX: IC_50_ = 0.018 ± 1.83 μM)	MSL	[59]
FZU-0038–056	Bcl-2↓ Mcl-1↓ XIAP↓	Induce apoptosis	HCC1806, HCC1937 (IC_50_ = 3 − 6 μM)	BL2, BL1	[60]
Oleandrin	Bcl-2↓ Bax↑ Bim↑	Induce apoptosis	MB-MDA-231 (IC_50_ = 24.62 nM)	MSL	[62]
(-)-Sativan (SA)	Bcl-2↓ Bax↑ Programmed cell death ligand 1 (PD-L1) ↓	Induce apoptosis	MDA-MB-231 (IC_50_ = 38.39 μM) BT549 (IC_50_ = 27.65 μM)	MSL, M	[63]
Cardamonin (CD)	Bcl-2↓ Bax↑ Cyt-c↑ caspase-3↑ PARP↑	Induce apoptosis	MDA-MB-231 (IC_50_ = 10 − 20 μM), BT-549 (IC_50_ = 0 − 10 μM)	MSL, M	[64]
Artesunate	Bcl-2↓Heat shock protein 70 (HSP70) ↓ caspase-9↑	Induce apoptosis	4T1 (IC_50_ = 52.41 μM)		[65]
Tetramethoxychalcone (TMC)	Bcl-2↓ Bax↑	Induce apoptosis	MDA-MB-231 (IC_50_ = 8.696 μM), BT549 (IC_50_ = 14.28 μM)	MSL, M	[66]
Trifluridine (TFT)	Bcl-2↓ Bax↑ caspase-7↑	Induce apoptosis	MDA-MB-231, BT-549, Hs578T (IC_50_ = 10 − 20 μM)	MSL, M	[67]
CCT020312	Bcl-2↓ Bax↑ PARP↑	Induce apoptosis	MDA-MB-453 (IC_50_ = 5 − 6 μM), CAL-148 (IC_50_ = 8 − 10 μM)	MSL, LAR	[68]
CH5126766	Bcl-2↓ Bcl-xl↓	Induce apoptosis	MDA-MB-231 (IC_50_ = 3 − 10 μM)	MSL	[69]
Physciosporin (PHY)	Bax↑ Bcl-xl↓	Induce apoptosis	MDA-MB-231 (IC_50_ = 27.3 μM)	MSL	[70]
CPSI-1306	ROS↑ Cyt-c↑	Induce apoptosis	MDA-MB-468 (IC_50_ = 0.84 μM); MDA-MB-231 (IC_50_ = 1.16 μM)	BL1, MSL	[73]
Berberine (BBR)	Cyt-c↑ caspase-9↑	Induce apoptosis	BT549 (IC_50_ = 16.575 ± 1.219 μM); MDA-MB-231 (IC_50_ = 18.525 ± 6.139 μM)	M, MSL	[75]
Budlein A methylacrylate (BAM)	NF-κB↓	Induce apoptosis	MDA-MB-231 (IC_50_ = 1.55 μM); MDA-MB-453 (IC_50_ = 1.75 μM); MDA-MB-436 (IC_50_ = 4.53 μM)	MSL, LAR	[79]
Icariin	SIRT6↑ NF-ΚB↓	Induce apoptosis	MDA-MB-231 (IC_50_ = 10 − 15 μM); MDA-MB-453 (IC_50_ = 5 − 10 μM)	MSL, LAR	[80]
Crambescidin 800 (C800)	Akt↓ NF-κB↓ MAPK↓	Induce apoptosis	SUM159PT (IC_50_ = 3.42 ± 0.07 μM); MDA-MB-231 (IC_50_ = 5.00 ± 0.57 μM); SUM149PT (IC_50_ = 6.02 ± 0.14 μM)	MSL, BL2	[81]
ACT001	NF-κB↓	Induce apoptosis	4T1 (IC_50_ = 43.2 μM)		[82]
Cedrelone acetate	NF-κB↓ EGFR↓ Akt↓	Induce apoptosis	MDA-MB-231 (IC_50_ = 1.28 ± 0.04 μM)	MSL	[83]
Ginsenoside panaxatriol (GPT)	NF-κB↓ ERK↓	Induce apoptosis	MB231-PR (IC_50_ = 21.39 μM)	MSL	[85]
Rg3	NF-κB↓	Induce apoptosis	MDA-MB-231, MDA-MB-453, BT-549 (IC_50_ = 20 − 25 μM)	MSL, LAR, M	[86]
Oprozomib	NF-κB↓	Induce apoptosis	MDA-MB-231 (IC_50_ = 0.079 μM); BT-549 (IC_50_ = 0.05 μM)	MSL, M	[87]
Carfilzomib (CARF)	NF-κB↓	Induce apoptosis	MDA-MB-231 (IC_50_ = 0 − 50 nM); MDA-MB-468 (IC_50_ = 50 − 100 nM)	MSL, BL1	[88]
DCC-2036	PI3K/Akt-NF-κB↓	Induce apoptosis	MDA-MB-231 (IC_50_ = 3.3 μM); HS-578 T (IC_50_ = 3.7 μM)	MSL	[89]
Aurantoside C (C828)	Akt/mammalian target of rapamycin (mTOR) ↓ NF-κB↓ P38 MAPK↑ Stress-activated protein kinase (SAPK)/JNK↑	Induce apoptosis	SUM159PT (IC_50_ = 0.56 ± 0.01 μM); MDA-MB-231 (IC_50_ = 0.61 ± 0.01 μM); SUM149PT (IC_50_ = 0.81 ± 0.02 μM)	MSL, BL2	[91]
KHF16	NF-κB↓	Induce apoptosis	MDA-MB-231 (IC50 = 6.8 μM); MDA-MB-468 (IC_50_ = 9.2 μM); SW527 (IC_50_ = 5 − 6 μM)	MSL, BL1	[92]
Noscapine	NF-κB↓	Induce apoptosis	MDA-MB-231 (IC_50_ = 20 μM)	MSL	[93]
SC-60	SHP-1/STAT3↓	Induce apoptosis	MDA-MB-231 (IC_50_ = 0.9 − 1.1 μM); MDA-MB-468 (IC_50_ = 1.8 − 2.0 μM); HCC1937 (IC_50_ = 3.9 − 4.1 μM)	MSL, BL1	[99]
SC-43	SHP-1/STAT3↓	Induce apoptosis	MDA-MB-231 (IC_50_ = 1.18 μM); MDA-MB-468 (IC_50_ = 1.3 μM); HCC1937 (IC_50_ = 1.72 μM)	MSL, BL1	[100]
SG-1721	STAT3↓	Induce apoptosis	MDA-MB-468 (IC_50_ = 6.90 μM)	BL1	[101]
Compound 15d	LIFR-JAK-STAT3↓	Induce apoptosis	MDA-MD-231 (IC_50_ = 3.83 ± 0.27 μM)	MSL	[102]
Ilamycin C	IL-6/STAT3↓	Induce apoptosis	MDA-MD-231 (IC_50_ = 7.26 μM) ; BT549 (IC_50_ = 6.91 μM)	MSL, M	[103]
Osthole	STAT3↓	Induce apoptosis	MDA-MD-231 (IC_50_ = 90.66 μM); BT549 (IC_50_ = 77.19 μM); MDA-MD-468 (IC_50_ = 70.65 μM)	MSL, M, BL1	[104]
Bt354	STAT3↓	Induce apoptosis	MDA-MB-435 (IC_50_ = 6.5 μM); MDA-MB-231 (IC_50_ = 7.2 μM)	MSL,	[105]
Primaquine	EGFR/STAT3↓	Induce apoptosis	MDA-MB-231 (IC_50_ = 81.2 μM)	MSL	[106]
Eupalinolide J (EJ)	STAT3↓	Induce apoptosis	MDA-MB-231 (IC_50_ = 3.74 ± 0.58 μM); MDA-MB-468 (IC_50_ = 4.30 ± 0.39 μM)	MSL, BL1	[107]
LLY17	IL-6/STAT3↓	Induce apoptosis	MDA-MB-468, MDA-MB-231, SUM159, BT-549	MSL, BL1, M	[108]
Pulvomycin	STAT3↓	Induce apoptosis	MDA-MB-231 (IC_50_ = 2 − 3 μM)	MSL	[109]
APR-246	p53↑	Induce apoptosis	MDA-MD-468, BT549 (IC_50_ = 2 − 4 μM); BT20 (IC_50_ = 4 − 6 μM)	BL1, M	[116, 117]
CX-5461	p53↑	Induce apoptosis	Hs578T (IC_50_ = 9.2 μM); BT549 (IC_50_ = 1.7 μM); MDA-MD-231 (IC_50_ = 1.6 μM); SUM159PT (IC_50_ = 2.0 μM)	M, MSL	
COTI-2	p53↑	Induce apoptosis	Hs578T (IC_50_ = 21.3 ± 5.4 nM); MDA-MD-468 (IC_50_ = 71.1 ± 15.1 nM); MDA-MD-231 (IC_50_ = 59.1 ± 4.9 nM); BT549 (IC_50_ = 2.5 ± 0.6 nM)	BL1, MSL, M	[118]
PK11007	p53↑	Induce apoptosis	MDA-MD-468 (IC_50_ = 2.8 ± 0.3 μM); HCC1937 (IC_50_ = 6.8 ± 3.2 μM); HCC1143 (IC_50_ = 2.3 ± 0.3 μM); BT549 (IC_50_ = 3.4 ± 0.6 μM); Hs578T (IC_50_ = 5.3 ± 0.7 μM)	BL1, M, MSL	[119]
Compound 5i	p53↑ MDM2↓	Induce apoptosis	MDA-MB-231 (IC_50_ = 3.5 ± 1.0 μM)	MSL	[120]
Ruthenium (II)/allopurinol complex	p53↑	Induce apoptosis	MDA-MB-231 (IC_50_ = 9.1 ± 0.9 μM)	MSL	[121]
Resveratrol (RSV)	p53↑ Polymerase (DNA) delta 1 (POLD1) ↓	Induce apoptosis	MDA-MB-231 (IC_50_ = 50 μM)	MSL	[122]
Ziyuglycoside I	p53↑ p21WAF1↑	Induce apoptosis	MDA-MB-231 (IC_50_ = 13.96 μM)	MSL	[123]
α-Conidendrin	p53↑ p21↑	Induce apoptosis	MDA-MB-231 (IC_50_ = 3.5 ± 1.0 μM)	MSL	[124]
I-7ab	p53↑	Induce apoptosis	MDA-MB-231 (IC_50_ = 35.48 μM); BT-20 (IC_50_ = 38.02 μM)	MSL	[125]
Ciprofloxacin	p53↑	Induce apoptosis	MDA-MB-231 (IC_50_ = 0.03 μM)	MSL	[126]

^*^↓, decrease/inhibition; ↑, increase/activation

### Targeting Bcl-2 family

Bcl-2 belongs to a growing family of proteins that regulated a unique programmed cell death, namely apoptosis. The Bcl-2 family proteins were central regulators of cell death, which played an important regulatory role in cell apoptosis [40]. The Bcl-2 family proteins regulated the permeability, stability and integrity of mitochondrial outer membrane (MOM), which was especially vital for the release of cytochromic c and the activation of downstream factors [41]. The Bcl-2 family was mainly divided into pro-apoptotic proteins (e.g., Bax, Bak) and anti-apoptotic proteins (e.g., Bcl-2, B-cell lymphoma-extra-large (Bcl-xl)). The balance of proapoptotic protein and anti-apoptotic protein was the key to determine whether cell apoptosis would occur [42–44]. Statistically, Bcl-2 antiapoptotic proteins showed abnormal expression in various malignancy, including 70% of breast cancers, 80% of B-cell lymphomas and other forms of cancer [45]. We conclude that several small molecular compounds can induce TNBC cells apoptosis by modulating the biological functions of Bcl-2 family-related proteins.

Novel spirooxindole is reported to have the anticancer effect of them on TNBC cell lines [46]. In the TNBC MDA-MB-231 cells, spirooxindole 6e, with a half maximal inhibitory concentration (IC_50_) value of 6.40 μM, was identified as the most potent compound among them. It could induce apoptosis in MDA-MB-231 cells through suppression of Bcl-2 protein, upregulation of Bax protein, as well as the activation of caspase-3 [46]. In addition, the concept of molecular hybridization was used and combined the pharmacodynamic elements of isatin and phthalazine or quinazoline in a chemical framework through a hydrazine linker to synthesize a novel 5-chloro-3–(2–(4–(4-chlorophenyl)phthalazin-1-yl)hydrazono)indolin-2-one (compound 10 g) targeting TNBC [47]. Compound 10 g was the most active hybrid with an IC_50_ value of 12.0 ± 0.13 μM and induced apoptosis through the enhanced expression of Bax, the reduced expression of Bcl-2 and the activated level of caspase-3,9 [47].

Sophoraflavanone G (SG) could inhibit Bcl-2 and Bcl-xl expressions, significantly stimulate Bax expression and inhibit the mitogen-activated protein kinase (MAPK) pathway, resulting in the induction of apoptosis as well as the inhibition of the migration and invasion [48]. IIamycin E, a natural product extracted from deep sea-derived Streptomyces atratus, could effectively suppress the proliferation and arrest G1/S cell cycle of HCC1937 and MDA-MB-468 cells. Meanwhile, IIamycin E induced apoptosis via downregulating Bcl-2 expression [49]. The combination of bioactive compounds exerted an effectively synergistic anticancer activity. Gallic acid (GA) and curcumin (Cur), as naturally plant derivatives, had been reported to have potently exerted anticancer effects through induction of apoptosis [50]. The combination of GA and Cur demonstrated a more significant promotive effect on Bax expression and caspase-3 cleavage than single compound utilized alone. In the meantime, the combination of GA and Cur could also decrease Bcl-2 expression dramatically. These regulatory activities led to a more essential proapoptotic effect of the co-treatment than these reagents used separately [50].

Natural compound derivatives have gradually been becoming a new source for the discovery of anti-TNBC drug candidates. KYZ3 (7-((4-fluorobenzyl)oxy)-2-methyl-2,3-dihydronaphtho[1,2-b].furan-4,5-dione), a cryptotanshinone derivative, exhibited approximately 22–24-fold higher antitumor activity against the MDA-MB-231 cells than its parent compound cryptotanshinone [51]. KYZ3 inhibited signal transducer and activator of transcription 3 (STAT3) phosphorylation, leading to the inhibition of STAT3-transcriptionally activated oncogenes, including Bcl-2. Also, KYZ3 could increase the level of Bax, ultimately leading to the increase in TNBC apoptotic cells [51]. ((1aR,7aS,10aS,10bS,E)-1a-Methyl-8-methylene-9-oxo-1a,2,3,6,7,7a,8,9,10a,10b-decahydrooxireno[2,3':9,10].cyclodeca[1,2-b].furan-5-yl)methyl (E)-3-(2,6-dimethoxyphenyl)acrylate (Compound 7d) was a parthenolide derivative, which could inhibit proliferation in TNBC cells via apoptosis induction through increasing the level of Bax and Bcl-2 interacting mediator of cell death (Bim) protein and promoting the cleavage of caspase-9. Moreover, Compound 7d could cause G1 phase arrest [52]. Furthermore, a new derivative of SMBA1 (Bax activators), CYD-4-61, was found to enhance the anti-proliferation activity of TNBC cell lines. CYD-4-61 was also reported to activate Bax protein, promote the release of Cyt-C and boost the cleavage of PARP-1 and caspase-3, thus inducing breast cancer cells apoptosis [53].

Combining some chemotherapeutic drugs with natural compound could not only improve the effectiveness of cancer treatment, but also reduced the toxicity and side effects of the chemotherapeutic drugs [54]. Doxorubicin (Dox) was an anthracycline antibiotic and a broad-spectrum anticancer agent; as a cytotoxic anthracycline antibiotic, it is often used as tumor chemotherapy agent for MOA during inhibition of topoisomerase and DNA replication [55]. Although Dox was effective in the treatment of TNBC, its actual use was limited due to side effects including cardiotoxicity [56]. Combination of Dox and small-molecule compounds to reduce the Dox dose could minimize the toxic and side effects [54, 57]. Arctigenin (ATG) exhibited its own anticancer activity, and when combined with Dox, ATG could enhance the cytotoxic effect of Dox on MDA-MB-231 cells. ATG and Dox co-treatment was induced by downregulating expression of Bcl-xl and Bcl-2, through non-major (or off-target) drug effects by promoting the translocalization of Bax to mitochondria, thereby destroying mitochondrial integrity [58]. In addition, coralline, a heterocyclic analog, combined with paclitaxel (PTX) had a synergistic effect on the inhibition of proliferation and migration of TNBC cells without any toxic effect on normal cells. The co-treatment could promote cell apoptosis by suppressing Bcl-2 and increasing Bax [59]. FZU-0038-056, a tetrahydro-β-carboline (THβC) skeleton derivative, was reported to induce TNBC cell lines apoptosis via enhancing the cleavage of caspase-3 and reducing Bcl-2, X-linked inhibitor of apoptosis protein (XIAP), and myeloid cell leukemia-1 (Mcl-1) proteins. Moreover, when it used together with cisplatin, the antitumor activity could be efficiently strengthened [60]. In addition, the study had shown that BCL2 inhibitor ABT199 is generally considered to be effectively only for Bcl-2-dependent cancers, but when combined with cisplatin, it could inhibit TNBC cells viability with less side effects [61]. Combinational drug therapy is a viable and effective strategy for cancer treatment. Apart from the compounds mentioned above, other small-molecule compounds that induce apoptosis of TNBC cells by regulating Bcl-2 family proteins are also summarized in Table 1 [62–70].

### Targeting Apaf-1 and Cyt-C

Cyt-C is a type of hemoglobin involved in the mitochondrial electron transport chain, which played an important role in REDOX and energy metabolism [71]. Meanwhile, Cyt-C is a crucial material in mitochondria to start the process of apoptosis. In the presence of deoxyadenosine triphosphate (dATP) and adenosine triphosphate (ATP), Cyt-C is released from mitochondria and bound to Apaf-1 to form a poly-complex, which in turn activated the caspase cascade and thus induced apoptosis [72].

Overexpression of macrophage migration inhibitory factor (MIF) has been an important prognostic factor in breast cancer by regulating tumor initiation, aggressiveness and progression. A recent report showed that MIF was overexpressed in TNBC; CPSI-1306 as a MIF inhibitor was found to decrease TNBC tumor growth and metastasis both in vitro and in vivo. It could increase the ROS level in TNBC cells, promote the release of Cyt-C and apoptosis-inducing factor (AIF) from mitochondria and thus induce cell apoptosis [73, 74]. Moreover, a natural isoquinoline alkaloid, berberine (BBR), was reported to induce caspase-9-dependent apoptosis by triggering the release of Cyt-C from mitochondria and downregulating Bcl-2. It also suppressed the tumor growth in TNBC xenograft mice [75] (Table 1).

### Targeting NF-κB pathway

NF-κB is a transcription factor of Rel family proteins, which was widely involved in a variety of cellular activities, such as cell cycle, cell proliferation, apoptosis, migration and invasion [76, 77]. In addition, NF-κB was associated with cancer initiation, metastasis and resistance. Particularly in TNBC, the abnormal activation of NF-κB was more frequent, which influenced the expression of its downstream signaling targets [78]. Therefore, selectively targeting NF-κB and its downstream signaling might be a promising therapeutic approach for the treatment of TNBC.

Budlein A methylacrylate (BAM), as an active compound isolated from *Helianthus genus* plant, showed selective cytotoxicity against TNBC cell lines without any toxic effect on normal cells [79]. BAM inhibited the activity of inhibitor kappa B kinaseβ (IKKβ) and exportin 1 (XPO-1) and then inhibited the NF-κB pathway, leading to TNBC cell apoptosis. Besides, the findings from the in vivo study suggested that it could decrease tumor growth [79]. Icariin was a prenylated flavonol glycoside, which had potent properties in various types of cancers. Icariin could upregulate silent information regulator 6 (SIRT6) expression to inhibit the activation of NF-κB pathways, thereby triggering apoptosis in TNBC cells [80]. Moreover, it also suppressed the tumor growth and pulmonary metastasis in both MDA-MB-231 and 4T1 mouse model [80]. Similarly, crambescidin 800 (C800), a guanidine alkaloid isolated from sponge, could decrease the phosphorylation of Akt, NF-κB and MAPK, resulting in apoptosis in TNBC cells [81]. A novel derivative of sesquiterpene lactone, ACT001, was found to suppress tumor angiogenesis and the accumulation of myeloid-derived suppressor cells (MDSCs) of 4T1 tumor-bearing mice, by inhibiting the activity of NF-κB pathway and successively inducing apoptosis [82]. Cedarone acetate was a compound obtained by acetylation modification of original cedarone molecule; when compared with the original molecule, it could enhance the cytotoxic activity and induce apoptosis through downregulating the level of NF-κB and matrix metallopeptidase 9 (MMP9) [83].

DOX and PTX were the most widely used chemotherapeutic drugs in the treatment of TNBC [84], but their high-dose use would have inherent drug resistance and serious side effects [54]. NF-κB could improve the resistance of chemotherapeutic drugs to TNBC cells by regulating anti-apoptotic pathways. The combination treatment of ginsenoside panaxatriol (GPT) and PTX could inhibit the tumor cells growth and induce apoptosis of TNBC cells resistant to PTX by suppressing interleukin-1 receptor-associated kinase 1 (IRAK1)-mediated NF-κB and ERK pathways [85]. In addition, GPT could increase the sensitivity of TNBC PTX-resistant cells [85]. Meanwhile, it was reported that ginsenoside Rg3 could promote the cytotoxicity and apoptosis of PTX on TNBC by reducing the expression of NF-κB [86]. Oprozomib and carfilzomib were both proteasome inhibitors, which could sensitize TNBC cells to DOX treatment and induce apoptosis by suppressing the activation of NF-κB and JNK/p38 MAPK phosphorylation [87, 88]. DCC-2036 was reported to exert an inhibitory effect on TNBC cells proliferation, migration and invasion, ultimately inducing apoptosis. It targeted Anexelekto (AXL)/MET to inhibit the PI3K/Akt-NF-κB pathway and epithelial–mesenchymal transition (EMT) [89, 90]. In vivo, DCC-2036 could suppress the growth and metastasis of tumor-burden mice. Besides, when DCC-2036 combined with cisplatin or lapatinib, the co-treatment showed a notable synergistic effect on TNBC [89]. In addition to compounds mentioned above, remaining small-molecule compounds targeting NF-κB to trigger apoptosis on TNBC cells are also collected [91–93] (Table 1).

### Targeting STAT3 pathway

STAT3, which is an essential intracellular signal transduction protein, can participate in the regulation of cancer cell proliferation, differentiation, apoptosis and invasion by acting on downstream related genes [94, 95]. STAT3 was activated in lung, prostate and breast cancer. STAT3 expression level is significantly higher in TNBC than that in other breast cancers and normal tissues [96, 97]. Several small-molecule compounds could induce cancer cells apoptosis to exhibit anticancer properties by suppressing the STAT3 phosphorylation process, which was considered as one of the crucial targets for TNBC treatment [98].

Sorafenib analogue, SC-60, has been reported to reduce TNBC cell viability and induced apoptosis by downregulating phosphorylated STAT3 expression in both a dose- and time-dependent manner. In addition, the combination of SC-60 and docetaxel synergistically enhanced the anticancer effect by inhibiting the SHP-1/STAT3 pathway [99]. Another sorafenib analogue, SC-43, was reported to block STAT3 signaling to increase the sensitivity of cancer cells to chemotherapeutic drugs like docetaxel. SC-43 showed tumor growth inhibition and apoptosis inducing by suppressing the SHP-1-dependent STAT3 expression [100]. SG-1721, a (-)-galiellalactone analogue, was found to inhibit the growth of TNBC cells. It could promote cells apoptosis via suppressing the nuclear translocation and DNA binding of STAT3, as well as reduce the expression of carcinogenic proteins such as Bcl-2, Cyclin D1 and MMP-2. In in vivo experiment, SG-1721 could significantly inhibit the growth of breast xenograft tumor. Moreover, when SG-1721 was combined with radiotherapy, TNBC cells were sensitized to radiation and apoptotic effect was enhanced. It could be a potential agent that targets STAT3 to treat TNBC [101]. Compound 15d as JAK/HDAC dual inhibitor exhibited the antiproliferative and proapoptotic activities by suppressing the activation of LIFR-JAK-STAT signaling and attenuate the drug resistance in tumor cells [102]. Ilamycin C, a new compound isolated from Streptomyces atratus SCSIO ZH16, exerted a strong cytotoxic activity against TNBC cells. It promoted cell apoptosis by inhibiting IL-6-induced STAT3 phosphorylation and suppressed TNBC cells migration and invasion through MMP2/MMP9/vimentin/fascin [103]. A growing body of research suggests that inhibition of STAT3 pathway has been considered as a novel therapeutic strategy to treat TNBC. Other small-molecule compounds target STAT3 to trigger apoptosis on TNBC [104–109] (Table 1).

### Targeting p53

p53 is an essential tumor suppressor protein, which could regulate diverse cellular processes, including cell apoptosis, DNA repair, cell cycle arrest, etc. [110]. Activating p53 protein could increase the sensitivity of cancer cells to DNA damage factors, so that cells with DNA damage could not enter the replication cycle, and then went to apoptosis [111]. P21, as a target gene of p53 protein, is involved in cell growth arrest and inhibits cell cycle progression by inhibiting cyclin B/Cdc2 through mitosis. Upregulation of p21 acts as the inhibitor of cell cycle dynamics [112]. Furthermore, more than 80% of TNBC patients had p53 mutation, and the mutant p53 protein could effectively promote the malignant transformation of cells, activate other oncogenes and lead to the occurrence of tumors [113, 114]. In recent years, some small-molecule compounds could reactivate the mutant p53 protein and restore it to the wild-type conformation, which made targeting the mutant p53 protein becoming one of the most attractive targets for treating TNBC [115].

APR-246 as a p53 activator could inhibit cell proliferation and migration and induce apoptosis in a p53 mutant-dependent manner of TNBC cells [116]. Recently, the study found that co-treatment of APR-246 and CX-5461, an RNA polymerase I inhibitor, could also significantly inhibit TNBC cells growth and induce apoptosis, which was caused by DNA damage [116, 117]. Furthermore, a third-generation thiosemicarbazone, COTI-2, targeted the p53 protein to upregulate the expression of apoptosis effector genes, such as Bcl-2 binding component 3 (BBC3) and phorbol-12-myristate-13-acetate-induced protein 1 (PMAIP1), leading to the induction of apoptosis in BT549 and Hs578t cells. COTI-2 could convert the mutant p53 protein into wild-type p53 to exhibit anticancer activity [118]. In general, the IC_50_ of COTI-2 was lower than that of APR-246, suggesting that COTI-2 was more active in inhibiting cell proliferation than APR-246 in TNBC cell lines [116, 118]. In addition, PK11007 was a mild mercaptan alkylation agent, which could inhibit cell growth and induce apoptosis in TNBC cells by upregulating of p53 protein. In addition, PK11007 combined with cisplatin could synergistically inhibit the growth of TNBC cell lines [119].

There are some natural and semisynthetic compounds targeting p53 protein to induce apoptosis of TNBC cell lines. In addition, it was found that a series of spirotriazoline oxindoles had been synthesized and 5-bromo-4'-(3-chlorophenyl)-20,50-diphenyl-20,40-dihy-drospiro[indoline-3,3'-[1, 2, 4].triazol].-2-one (compound 5i) was identified as the compound with the most significant inhibitory effect on MDA-MB-231 cell [120]. After compound 5i treated MDA-MB-231 cells, the expression of p53 was upregulated and the expression of murine double minute 2 (MDM2) was downregulated, and then compound 5i could increase the expression of tumor suppressor proteins, arrest cell cycle at G0/G1 phase, inhibit cell proliferation and eventually lead to cell apoptosis [120]. Moreover, it was also found that organometallic complexes, like ruthenium (II)/allopurinol complex, had been shown to have significant cytotoxicity against TNBC cells [121]. In contrast, ruthenium (II)/allopurinol complex binds to tumor cells to cause DNA damage and upregulate p53 protein, then causing the overexpression of bim, beclin-1 and caspase-3, and ultimately induced cell apoptosis [121]. Additional small-molecule compounds target p53 protein to induce apoptosis in TNBC cells [122–126] (Table 1).

## Targeting autophagy-dependent cell death pathways with small-molecule compounds in TNBC

**Fig. 2:**
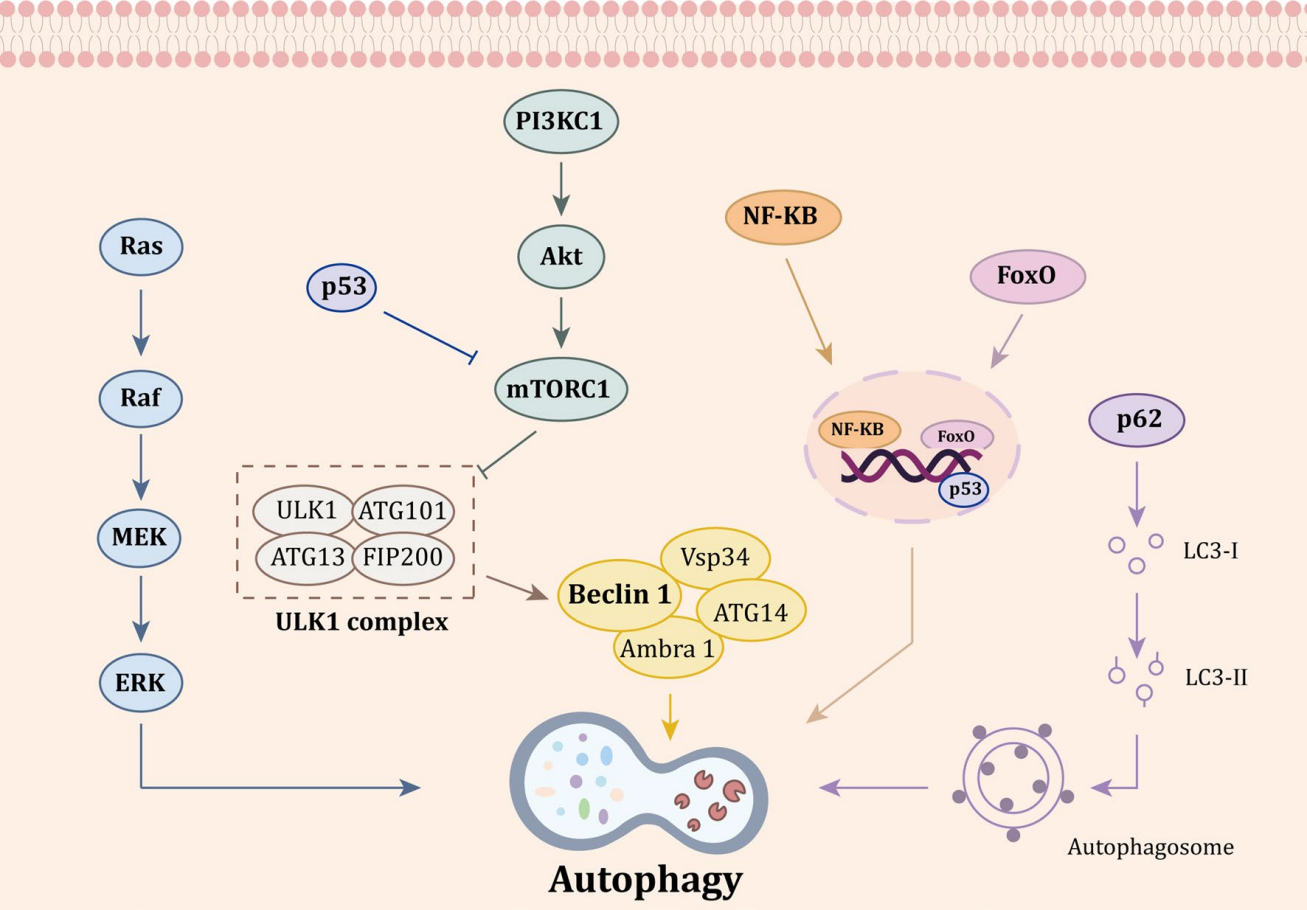
Core autophagy-dependent cell death signaling pathways in triple negative breast cancer (TNBC). Autophagy is a complex regulation process involving many upstream signaling pathways. Mammalian target of rapamycin (mTOR) is a negative regulator of autophagy, which is composed of mammalian target of rapamycin complex (mTORC) 1 and mTORC2. Among them, mTORC1 is the main autophagy regulator and phosphatidylinositol 3 kinase complex 1 (PI3KC1)-protein kinase B (Akt)-mTORC1 pathway inhibits the occurrence of autophagy. P53 pathway negatively regulates mTOR pathways to promote autophagy. When mTORC1 is inhibited, it can indirectly activate unc-51-like kinase 1 (ULK1) complex (including ULK1, autophagy associated protein (ATG) 101, ATG13, and focal adhesion kinase interacting protein of 200 kDa (FIP200)). ULK1 complex is closely related to Beclin1, and ULK1 can phosphorylate ATG14, which promotes the binding of Beclin1 to vacuolar protein sorting 34 (VPS34) and ultimately participates in the regulation of autophagy. Forkhead box O (FoxO) had been shown to regulate autophagy by transcriptional dependent mechanism. P62 can bind light chain 3 (LC3)-labeled autophagosomes to substrates, promote the combination of substrates and autophagosomes, and promote the occurrence of autophagy. Additionally, the inhibiting of Ras-Raf-MAPK pathway and NF-κB pathway could also regulate autophagy. Abbreviations: AKT: Protein kinase B; ATG: Autophagy associated protein; ERK: Extracellular signal-regulated kinase; FIP200: Focal adhesion kinase interacting protein of 200 kDa; FoxO: Forkhead box O; LC3: Light chain 3; MEK: Mitogen-activated protein kinase kinase; mTORC1: Mammalian target of rapamycin complex 1; PI3KC1: Phosphatidylinositol 3 kinase complex 1; ULK1: Unc-51-like kinase 1; Vps34: Vacuolar protein sorting 34

Autophagy is a process in which the excess proteins of damaged cellular organelles are degraded by lysosome in order to maintain cell homeostasis under stress [127]. Cells can recover energy and nutrients from autophagy degradation products, so that cells can maintain their own metabolism and enhance their tolerance to adverse stimulation [128]. Thus, autophagy can also be regarded as a self-protection mechanism of cells [129]. Autophagy has been becoming a new target of breast cancer treatment, but the role of autophagy in cancer is quite complex, which acts as a double-edged sword in the tumor treatment [130–132]. On the one hand, it can increase tumor cell autophagy activity, which contributes to programmed forms of cell death. On the other hand, autophagy may provide energy for tumor cell metabolism of maintaining cells survival (Fig. 2) [133, 134].

Autophagy is a complex regulation process involving many upstream signaling pathways. Mammalian target of rapamycin (mTOR) is a negative regulator of autophagy, which is composed of mammalian target of rapamycin complex (mTORC) 1 and mTORC2. Among them, mTORC1 is the main autophagy regulator and phosphatidylinositol 3 kinase complex 1 (PI3KC1)-protein kinase B (Akt)-mTORC1 pathway inhibits the occurrence of autophagy. p53 pathway negatively regulates mTOR pathways to promote autophagy. When mTORC1 is inhibited, it can indirectly activate unc-51-like kinase 1 (ULK1) complex (including ULK1, autophagy-associated protein (ATG101, mATG13 and focal adhesion kinase interacting protein of 200 kDa (FIP200)). ULK1 complex is closely related to Beclin1, and ULK1 can phosphorylate ATG14, which promotes the binding of Beclin1 to vacuolar protein sorting 34 (VPS34) and ultimately participates in the regulation of autophagy. Forkhead box O (FoxO) had been shown to regulate autophagy by transcriptional-dependent mechanism. P62 can bind light chain 3 (LC3)-labeled autophagosomes to substrates, promote the combination of substrates and autophagosomes, and promote the occurrence of autophagy. Additionally, the inhibiting of Ras-Raf-MAPK pathway and NF-κB pathway could also regulate autophagy.

Autophagy modulation can be served as a promising target for the development of anti-TNBC drugs, which had profound implications on breast cancer investigation [135, 136]. Several small-molecule compounds could alleviate or treat TNBC by changing the level of autophagy in tumor cells [137]. Moreover, autophagy had great potential in improving the therapeutic efficiency, overcoming chemotherapy resistance of breast cancer [138]. A variety of signaling pathways and target proteins had been implicated in autophagy regulation, including unc-51-like kinase 1 (ULK1) complex, PI3KCI-Akt-mTORC1, Ras-Raf-MAPKs, p53, p62, fork-head box O (FoxO), NF-κB, Beclin-1, etc. [139]. Then, we summarized the research progress of autophagy-related signaling pathways and their corresponding small-molecule compounds in the treatment of TNBC.

### Targeting the ULK1 complex

ULK1, a homologous protein of the yeast autophagy-associated protein 1 (ATG1) in mammals, is a serine/threonine protein kinase and a major regulator of autophagy initiation [140]. It interacted with focal adhesion kinase interacting protein of 200 kDa (FIP200), a protein in the adhesion spot kinase family, autophagy-related proteins mammalian autophagy-associated protein 13 (mAtg13) and Atg101 to form the ULK complex, which further activated the downstream autophagy signaling pathway and promoted the formation of autophagosomes [141]. ULK1, as a promoter of autophagy, also played different roles in different tumors. Downregulation of ULK1 expression was closely related to the progression of breast cancer and was accompanied by the decrease in autophagy level [142, 143]. Therefore, activation of ULK1-regulated autophagy cell death is a potential strategy for the treatment of TNBC [143]. There are some small-molecular compounds that directly or indirectly target ULK1 for TNBC therapy by regulating autophagy.

A small-molecular agonist, LYN-1604, was found to target ULK1 with a median effective concentration (EC_50_) value of 18.94 nm [144]. Based on site-directed mutagenesis and biochemical detection, three amino acid residues (LYS50, LEU53 and TYR89) were identified to be important for LYN-1604 binding and activation of ULK1. LYN-1604 was proved to induce TNBC cell death through autophagy via activating ULK1 and its complex. LYN-1604 could also activate ULK1 interactors including activating transcription factor 3 (ATF3), RAD21 and caspase-3, to induce cell death [144]. FL-411, a bromodomain 4 (BRD4) inhibitor, induced autophagy-associated cell death, which was dependent on the activation of the adenosine 5′-monophosphate-activated protein kinase (AMPK)–mammalian target of rapamycin (mTOR)–ULK1-modulated autophagic pathway [145]. In contrast, SBP-7455 as a ULK1/2 inhibitor could inhibit TNBC cells survival and proliferation by inhibiting starvation-induced autophagic flux. Moreover, SBP-7455, in combination with PARP inhibitor Olaparib, showed an enhanced effect on apoptosis in TNBC cells [146].

Autophagy had also been shown to play a key role in the chemotherapy resistance of TNBC [147]. Phloretin (PH), a dihydrochalcone flavonoid compound, could effectively inhibit the growth of TNBC cell by suppressing of autophagy through the downregulation of mTOR/ULK1 signaling pathway. Besides, PH overcomes resistance to chemotherapy drugs (4OH-Tamoxifen and DOX) by regulating autophagy in TNBC cells [148] (Table 2).

**Table 2:** Small-molecule compounds targeting autophagy in TNBC

Name in the literature and chemical structure	Target	Mechanism in RCD	Biological activity	TNBC subtype	References
LYN-1604	ULK1↑	Induce autophagy-dependent cell death	ULK1 (EC_50_ = 18.94 nm)	MSL	[144]
FL-411	AMPK-mTOR-ULK1↑	Induce autophagy-dependent cell death	MDA-MB-231 (IC_50_ = 3.27 ± 0.14 μM)	MSL	[145]
SBP-7455	ULK1/2↓	Inhibit autophagy-dependent cell death	ULK1 (IC_50_ = 13 nM)	BL1, MSL, M	[146]
Phloretin (PH)	mTOR/ULK1↓	Inhibit autophagy-dependent cell death	MDA-MB-231	MSL	[148]
SLLN-15	AURKA↓ AKT-mTOR↓	Induce autophagy-dependent cell death	MDA-MB-231, BT20 (IC_50_ = 10 − 25 μM)	MSL	[155]
Compound 9 m	mTOR↓	Induce autophagy-dependent cell death	mTOR (IC_50_ = 7 nM)	MSL	[156]
Hydroxychloroquine (HCQ)	Ras/Raf/ERK↓	Inhibit autophagy-dependent cell death	SUM190		[162]
Y29	AKT-MAPK↓	Induce autophagy-dependent cell death	MDA-MB-231 (IC_50_ = 630 nm)	MSL	[164]
CP-31398	wt-p53↑	Induce autophagy-dependent cell death	MDA-MB-231	MSL	[171]
PC3-15	p62↑	Inhibit autophagy-dependent cell death	EC_50_ = 13.50 μM	BL1, MSL	[174]
WX20120108	ROS-FoxO3↑	Induce autophagy-dependent cell death	MDA-MB-231 (IC_50_ = 14.37 ± 1.49 μM)	MSL	[178]
Alisol A	NF-κB↓ PI3K/AKT/mTOR↓	Induce autophagy-dependent cell death	MDA-MB-231 (IC_50_ = 10 − 20 μM)	MSL	[182]
Thymoquinone (TQ)	Beclin-1↓ LC3↓	Inhibit autophagy-dependent cell death	MDA-MB-231	MSL	[190]
Rhein 4F	Beclin-1↑ LC3-II/I↑	Induce autophagy-dependent cell death	MDA-MB-231 (IC50 = 12.80 ± 0.83 μM)	MSL	[192]

^*^↓, decrease/inhibition; ↑, increase/activation

### Targeting PI3KC1-Akt-mTORC1

The PI3KC1-Akt-mTORC1 signal transduction pathway was one of the central pathways regulating autophagy and was also involved in the regulation of tumor cell growth, proliferation and metabolism [149]. PI3K was a complex family which can be divided into PI3KC1, PI3KC2 and PI3KC3. PI3KC1 was involved in cell proliferation, insulin signal transduction, immune function and inflammatory response. Akt (serine/threonine kinase) was a protein kinase downstream of PI3K, which could activate and regulate multiple downstream targets [150, 151]. mTOR is a member of the PI3K family of protein kinases and existed in cells in the form of two complexes, mTORC1 and mTORC2. mTORC1 had an essential role in maintaining cellular homeostasis as a sensor of energy and nutrition in the cell [152, 153]. PI3KC1 activated Akt, which then directly phosphorylated and activated mTORC1 or indirectly activated mTORC1 by inhibiting tuberous sclerosis complex 1/2 (TSC1/2) and GTP-active Rheb, thereby inhibiting autophagy [154].

An increasing number of small-molecule compounds could induce TNBC cell death by targeting PI3KC1-Akt-mTORC1. A novel small molecule, SLLN-15, was shown to exhibit anticancer activity and inhibit the growth and proliferation of TNBC cells. These effects were associated with autophagy induction via decreasing the expression of aurora kinase A (AURKA) and blockade of AKT-mTOR signaling pathway [155]. Besides, 2-(4-(9-(6-aminopyridin-3-yl)-2-oxopyrazino[2,3-c].-quinolin-1(2H)-yl)piperidin-1-yl)acetonitrile (compound 9 m), a novel mTOR inhibitor, could induce the G0/G1 phase arrest of cell cycle and autophagy by inhibiting the phosphorylation of Akt, 4E-BP1 and S6. Besides, compound 9 m could significantly induce tumor regression in vivo experiment (Table 2) [156].

### Targeting Ras-Raf-MAPKs

MAPK is a signaling pathway that converted extracellular signals into intracellular signals in the form of a tertiary kinase cascade (MAPKKK-MAPKK-MAPK), among which Ras-Raf-MEK-ERK pathway had been studied most deeply [157]. More and more data indicated that MAPK (Ras-Raf-MEK-ERK) pathway was a vital target for TNBC treatment. Ras was a GTP binding protein, which activated Ras by binding to GTP [158], then phosphorylated Raf, causing downstream MEK/ERK activation, and then caused a series of physiological responses. Ras-Raf-MEK-ERK signaling pathway was not only widely involved in the regulation of multiple physiological processes such as cell growth, proliferation and apoptosis, but also involved in the regulation of autophagy and the promotion of autophagy-dependent death of tumor cells [159, 160]. This signaling pathway could also directly induce autophagy by upregulating the expression of autophagy-related proteins such as light chain 3 (LC3) and p62 [161]. Consequently, small-molecular compounds targeting Ras-Raf-MAPK pathway to induce breast cancer cells autophagy death was a promising approach for the treatment of TNBC.

Sometimes, the inhibition of autophagy could restrain the energy of cell metabolism and survival in cancer. HCQ as an autophagy inhibitor could suppress the cell proliferation and invasion and increase the sensitivity of breast cancer cells to 5-FU. HCQ could inhibit autophagy, and its inhibitory effect on SUM190 cells can be achieved by downregulating Ras-Raf-ERK pathway [162, 163]. Y29, a synthetic pyridine derivative, has markedly antiproliferative activity against TNBC cells. Y29 could induce autophagy by targeting platelet-derived growth factor receptor β (PDGFR-β), and it could enhance autophagy-related cell death through regulating AKT-MAPK pathway in MDA-MB-231 cells [164] (Table 2).

### Targeting p53

p53 protein not only induces apoptosis, but participates in autophagy as a critical regulator [165]. Recent reports showed that p53 played dual roles in the regulation of autophagy. The regulatory effect of p53 protein on autophagy was dependent on its intracellular localization [166]. In the nucleus, p53 protein induced autophagy and promoted cell death, while in the cytoplasm p53 inhibited autophagy, which could promote the survival of cancer cells in the occurrence and development of tumors [167]. During starvation or hypoxia, p53 activation is induced, which activates AMPK, further phosphorylates TSC1/TSC2, upregulates mTOR inhibitor phosphatase and tensin homologue deleted on chromosome ten (PTEN), and inhibits mTOR to induce autophagy [168].

High expression of mutant p53 protein was found in most TNBC, which is one of the causes of malignant cell proliferation [114]. Small-molecule compounds could target p53 mutant protein, induce cell autophagy and promote cancer cell death. For example, it was reported that small-molecule compound, CP-31398, could reactivate wild-type p53 protein to induce autophagy, thereby enhancing natural killer cell (NK) lysis of p53-mutated MB-MDA-231 cells [169, 170]. This finding suggested a new approach for future combination immunotherapy [171].

### Targeting p62, FoxO and NF-κB

p62, also called SQSTM1, is a receptor that could bind to ubiquitin and LC3 proteins, thus targeting autophagosomes and promoting the degradation of ubiquitin proteins [172]. During autophagy, p62 protein was continuously degraded in the cytoplasm, and p62 protein was continuously accumulated in the cytoplasm when autophagy was inhibited, so p62 could be used as a marker of autophagy activation [172, 173]. PC3-15, as a Schisandraceae triterpenoid compound, bound to the ubiquitination enzyme UbcH5b and suppressed the ubiquitination of p62, thereby inhibiting lapatinib-induced autophagy and increasing the sensitivity of TNBC cells to lapatinib therapy both in vitro and in vivo. These findings proved that UbcH5b-p62 axis was a potential therapeutic target for TNBC cell resistance [174].

FoxO is an essential autophagy regulator. FoxO in mammals included FoxO1, FoxO3, FoxO4 and FoxO6 subtypes, among which FoxO1 and FoxO3 had the most extensive effects [175, 176]. FoxO was involved in the regulation of cell proliferation, metabolism, survival and other processes by activating autophagy. FoxO regulated autophagy-associated with multiple signaling pathways, including AMPK, PI3K-Akt, etc. [175, 177]. Therefore, targeted therapy of autophagy genes could be a potential therapeutic strategy in TNBC breast cancer. WX20120108 was a novel IAP inhibitor with strong antitumor and pro-autophagic activity [178]. It also found that WX20120108 promoted the release of ROS to activate FoxO3, thereby inducing autophagy in MDA-MB-231 cell and HeLa cell [178].

In addition to regulating cell survival, apoptosis and inflammatory activation, NF-κB transcription factor was also involved in the regulation of autophagy [179, 180]. The regulatory relationship between NF-κB pathway and autophagy was complex. According to the different external environment, NF-κB could activate or inhibit autophagy, induce the body to produce protective or damage effect. Several small-molecule compounds could play a good therapeutic effect by acting on NF-κB signaling pathway to mediate autophagy [181]. Alisol A, a natural active compound of Alismatis rhizoma, could significantly suppress the proliferation and migration of TNBC cells when autophagy exerted an oncogenic effect. Alisol A induced MDA-MB-231 cells autophagy death by inhibiting both NF-κB and PI3K/AKT/mTOR pathways [182–184].

### Targeting Beclin-1

Beclin-1 (a homolog of yeast autophagy gene *ATG6/Vps30*, also known as BECN1) is one of the key autophagy regulatory proteins, which is involved in autophagosome membrane formation. *Beclin-1*, as an important tumor suppressor gene, was missing in most breast cancer patients, especially in TNBC [185, 186]. When the expression level of Beclin-1 was reduced, the autophagy activity of cells would also be inhibited, so that tumor cells become more aggressive. While *Beclin-1*, as a key gene for the formation of autophagosome initiation complex, was highly expressed in breast cancer, the proliferation and drug resistance of TNBC were dramatically restrained [163, 187–189]. Therefore, Beclin-1 could not only reduce the risk of breast cancer, but also affected the development of breast cancer by regulating autophagy.

Thymoquinone (TQ), a phenolic compound of Nigella sativa, was found to suppress the cell proliferation and invasion by inhibiting LC3, Beclin-1 and related oncogenic signaling (VEGF, MMPs) in TNBC cells, leading to the inhibition of oncogenic autophagy [190]. Rhein is a natural anthraquinone compound with significant antiproliferative and antimetastatic effects. However, due to poor bioavailability and lack of specific targets of this compound [191], Rhein was modified to obtain more efficient Rhein derivative 4F. Rhein 4F was reported to induce autophagy-dependent death of MDA-MB-231 cells through the upregulation of Beclin-1 and LC3-II/I and the downregulation of p62. Since the expression of apoptosis marker-caspase-3 was not affected by Rhein 4F, Rhein 4F-induced MDA-MB-231 cell death was mainly dependent on autophagy rather than apoptosis [192].

## Targeting other RCD subroutines with small-molecule compounds in TNBC

**Fig. 3:**
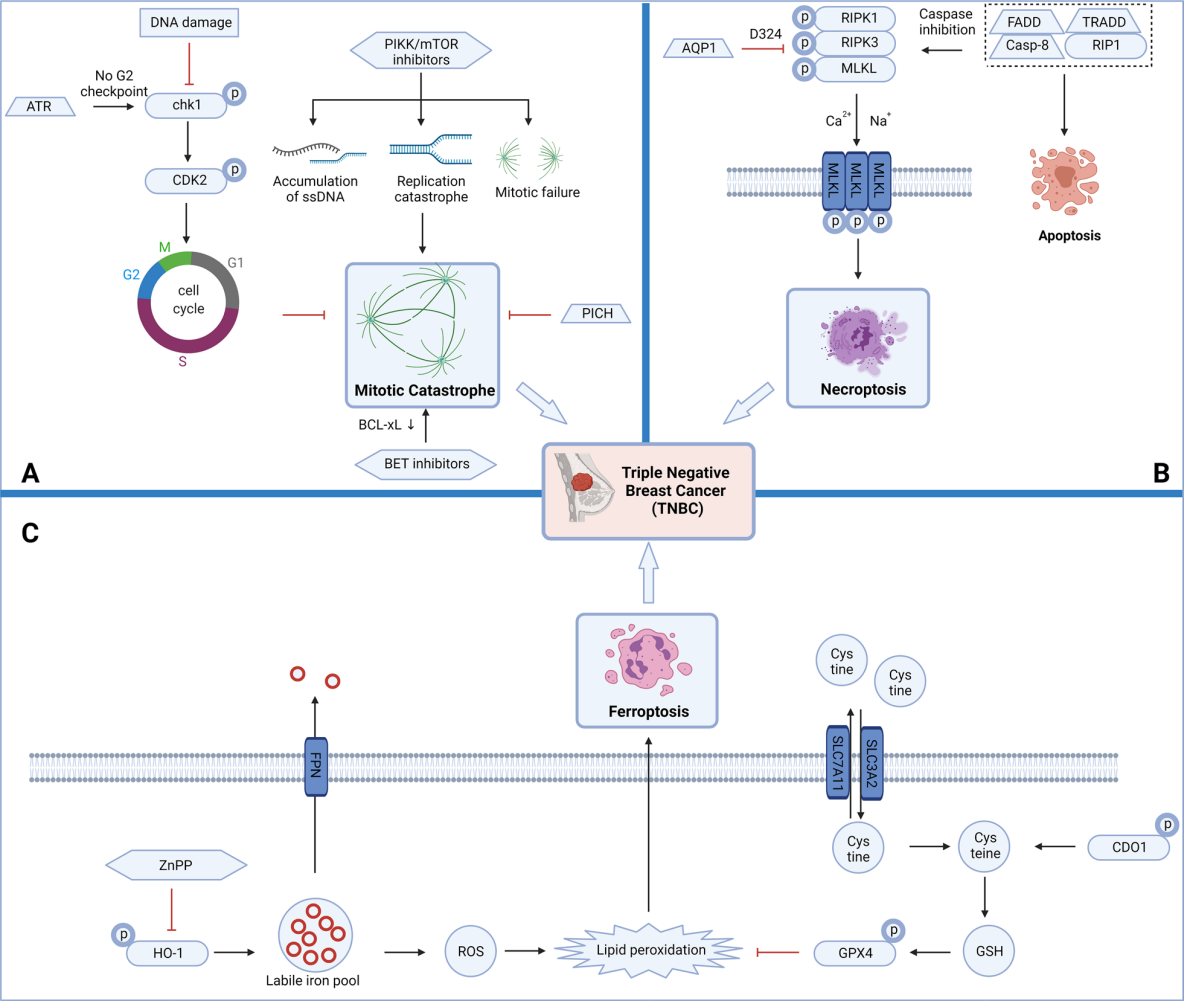
The key mitotic catastrophe, necroptosis and ferroptosis pathways in triple-negative breast cancer (TNBC). **a** DNA damage inhibits checkpoint kinase 1 (chk1) and cyclin-dependent kinase (CDK) 2 targets and then inhibits the recovery of cell cycle checkpoints, resulting in mitotic catastrophe. The rad3-related protein (ATR)-chk1 signaling pathway is activated in the absence of G2 checkpoints, restores S/G2 and G2/M cell cycle checkpoints and avoids the production of mitotic catastrophe. PI3K-like kinase (PIKK)/mammalian target of rapamycin (mTOR) inhibitors cause the accumulation of single-stranded deoxyribonucleic acid (ssDNA), replication catastrophe and mitotic failure, and ultimately lead to mitotic catastrophe. Polo-like kinase 1 (Plk1)-interacting checkpoint helicase (PICH) depletion can also lead to mitotic catastrophe. Bromodomain and extraterminal protein (BET) inhibitors eventually cause mitotic catastrophe by inhibiting B-cell lymphoma-extra-large (Bcl-xL). The production of the above mitotic catastrophe will eventually cause the death of TNBC cells; **b** Aquaporin1 (AQP1) can inhibit receptor-interacting protein (RIPK) 1/RIPK3/mixed lineage kinase domain-like (MLKL)-mediated necroptosis by binding to the D324 site of RIPK1. The fas associated via death domain (FADD)/TNFRSF1A associated via death domain (TRADD) complex depends on both RIPK1/caspase-8-mediated apoptosis and RIPK1/RIPK3/MLKL-mediated necroptosis. The production of the above necroptosis will eventually cause the death of TNBC cells; **c** Zn protoporphyrin IX (Znpp) inhibits the accumulation of unstable iron pools by inhibiting HO-1, reduces reactive oxygen species (ROS) levels and reduces ferroptosis caused by lipid peroxidation. Cystine enters and exits the cell membrane through solute carrier family 7 member 11 (SLC7A11)/solute carrier family 3 member 2 (SLC3A2), converts to cysteine, causes glutathione (GSH) levels to rise, activates Glutathione peroxidase 4 (GPX4) and inhibits ferroptosis caused by lipid peroxidation. The production of the above ferroptosis will eventually cause the death of TNBC cells

**Fig. 4:**
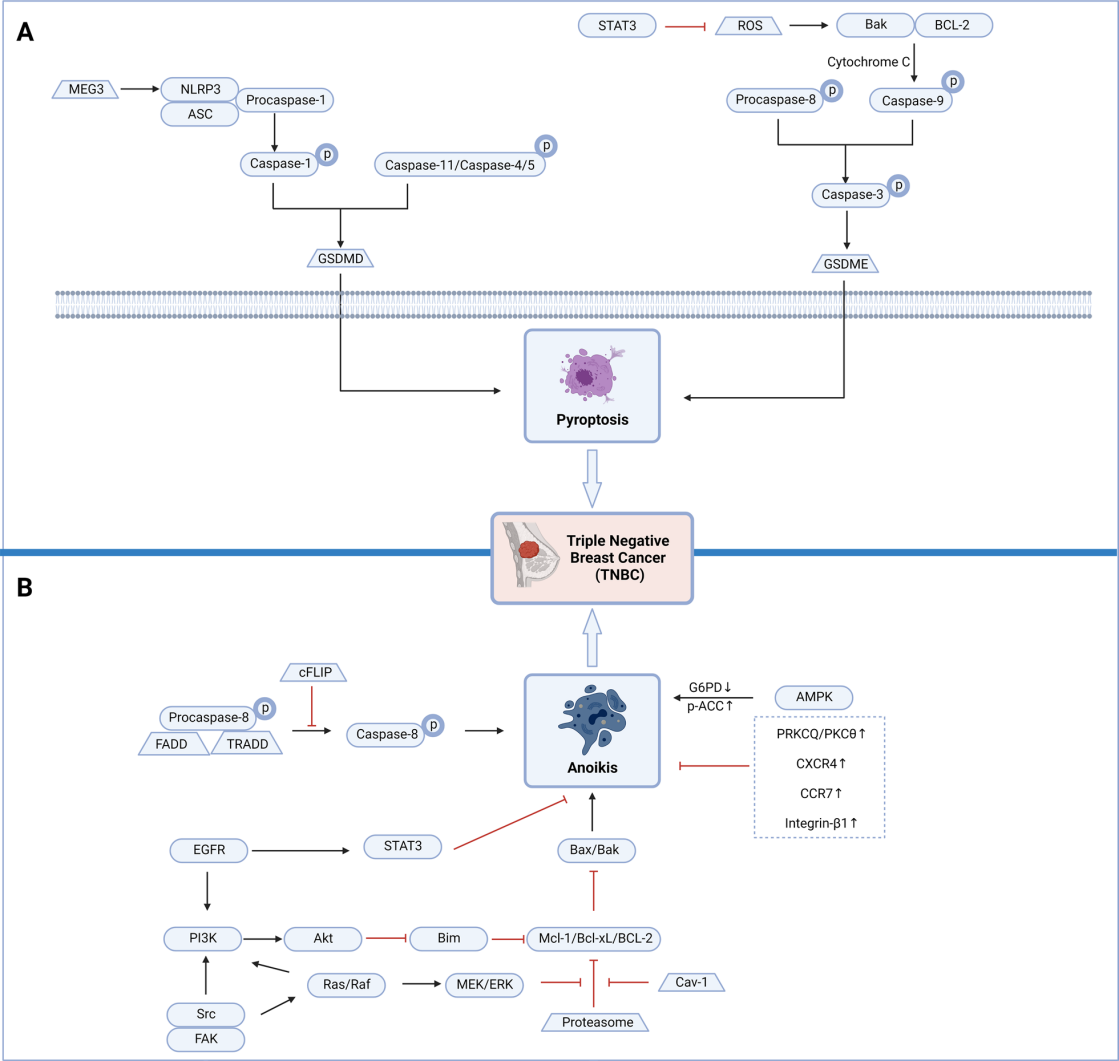
The key pyroptosis and anoikis pathways in triple-negative breast cancer (TNBC). **a** *Maternally expressed gene 3* (*MEG3*) activates the NLR family, pyrin domain containing 3 (NLRP3)/procaspase-1/apoptosis-associated speck-like protein containing a CRAD (ASC) complex, procaspase-1 is converted to caspase-1, and pyroptosis is induced via gasdermin D (GSDMD). In addition, caspase-11 and caspase-4/5 can also induce pyroptosis via GSDMD. Inhibition of mitochondrial signal transducer and activator of transcription 3 (STAT3) phosphorylation can increase reactive oxygen species (ROS) levels, activate bak and B-cell lymphoma 2 (Bcl-2) targets, activate caspase-9 and caspase-3 in the presence of cytochrome c (Cyt-c), and ultimately promote the cleavage of gasdermin E (GSDME), to transform apoptosis into pyroptosis. In addition, procaspase-8 can also activate caspase-3 to induce pyroptosis via GSDME. The above pyroptosis will eventually cause the death of TNBC cells. **b** cFLIP inhibits the production of anoikis by inhibiting the conversion of procaspase-8/fas associated via death domain (FADD)/TNFRSF1A associated via death domain (TRADD) complex to caspase-8. After epidermal growth factor receptor (EGFR) is activated, it inhibits the phosphorylation of Tyr705 on STAT3 and resists anoikis. EGFR and SRC/FAK activate the PI3K/Akt pathway, mediate late Bcl-2 interacting mediator of cell death (BIM) degradation, activate myeloid cell leukemia-1 (Mcl-1)/B-cell lymphoma-extra large (Bcl-xL)/Bcl-2, reduce Bax/Bak activity, and inhibit the production of anoikis. SRC/FAK also activates the Ras/Raf/MEK/extracellular signal-regulated kinase (ERK) pathway, relieves the inhibitory effect of proteasome on Mcl-1/Bcl-xL/BCL-2, reduces the activity of Bax/Bak, and inhibits anoikis. In addition, caveolin-1 (cav-1) can also restore the activity of Mcl-1/Bcl-xL/BCL-2 and ultimately inhibit anoikis. After adenosine 5′-monophosphate-activated protein kinase (AMPK) is activated, it can reduce glucose-6-phosphate dehydrogenase (G6PD) and increase phospho-acetyl-CoA carboxylase (p-ACC) and finally activate anoikis. In addition, the overexpression of protein kinase c theta (PRKCQ)/protein kinase C theta (PKCθ), C-X-C motif chemokine receptor 4 (CXCR4), C–C motif chemokine receptor 7 (CCR7) and integrin-β1 will inhibit anoikis. The production of the above anoikis will eventually cause the death of TNBC cells

Besides the classical apoptosis and autophagy-dependent cell death pathways, there are some other cell death measures belonging to RCD. According to the latest progress, RCD also includes mitotic catastrophe, necroptosis, ferroptosis, pyroptosis and anoikis, with their regulatory signaling pathways in TNBC (Table 3) (Figs. 3, 4).

**Table 3:** Small-molecule compounds targeting other RCD subroutines in TNBC

Chemical structure	Name in the literature	Target	Mechanism in RCD	Activity	TNBC subtype	References
	PTX	Microtubule inhibitor	Active mitotic catastrophe	MDA-MB-231 (IC_50_ = 5.78 μM) Hs578T (IC_50_ = 0.15 μM)	MSL	[199]
	JQ1	BET inhibitor	Active mitotic catastrophe	MDA-MB-231 (50 nM) HCC70 (500 nM) HCC1143 (200 nM)	BL1 MSL	[193, 205]
	SB218078	Chk1 inhibitor	Active mitotic catastrophe	HCC1937 (IC_50_ > 10 μM) IGRBr-11 (IC_50=_8.56 μM)	BL1 UNS	[208]
	Torin2	mTOR and PIKKs inhibitor	Active mitotic catastrophe	HCC1806 (GR_50_ = 0.08 μM) HCC70 (GR_50_ = 0.02 μM)	BL2	[214]
	KX-01	SRC and tubulin inhibitor	Active mitotic catastrophe	MDA-MB-231 (IC_50_ = 0.0446 ± 0.0009 μM) MDA-MB-468 (IC_50_ = 0.0613 ± 0.0017 μM) BT-549 (IC_50_ = 0.0467 ± 0.0019 μM)	BL1 M MSL	[215]
	erastin@FA-exo	Ferroptosis inducer	Active ferroptosis	MDA-MB-231 (IC_50_ = 10 μM)	MSL	[248]
As_4_O_6_	Tetraarsenic hexoxide (As_4_O_6_)	STAT3 inhibitor	Active pyroptosis	Hs578T (IC_50_ > 10 μM) MDA-MB-231 (IC_50_ = 5 μM)	MSL	[258]
	Cisplatin	Activation of *MEG3*/NLRP3/caspase-1/GSDMD Pathway	Active pyroptosis	MDA-MB-231 (IC_50_ = 9.952 nM)	MSL	[260]
	AEB071	PKCθ inhibitor	Active anoikis	MDA-231-Luc-D3H2LN (IC_50_ = 500 nM)		[268]
	Synthesized flavonoid derivative GL-V9	Glucose-6-phosphate dehydrogenase (G6PD)↓ Phospho-acetyl-CoA carboxylase (p-ACC) AMPK↑	Active anoikis	MDA-MB-231 (IC_50_ = 20 μM)	MSL	[271]
	Tubeimoside V (TBMS-V)	EGFR↑ ITGB1-FAK↑	Active anoikis	MDA-MB-231 (IC_50_ = 2.5 μM)	MSL	[272]
	Disulfiram/copper (DSF/Cu)	Calpain and decomposing vimentin in a Cu-dependent manner↑	Active anoikis	MDA-MB-231 (IC_50_ = 1 μM) Hs578T (IC_50_ = 1 μM)	MSL	[274]
	Salinomycin	STAT3 inhibitor	Active anoikis	MDA-MB-231 (IC_50_ = 10 μM, 72 h)	MSL	[286]
	POL5551	CXCR4 inhibitor	Active anoikis		MSL	[294]
	AL10	Sialyltransferase inhibitor	Active anoikis	MDA-MB-231 (IC_50_ = 10 μM)	MSL	[296]
	Berberine (BBR)		Active anoikis	MDA-MB-231 (IC_50_ = 10 μM)	MSL	[276]
	HPW-RX40	Integrin inhibitor	Active anoikis	MDA-MB-231 (IC_50_ = 15.2 μM)	MSL	[297]
	Archazolid A	V-ATPase inhibitor	Active and inhibit anoikis	MDA-MB-231 (10 nM)	MSL	[298]
	Narciclasine	Upregulate AMPK-ULK1 pathway	Active autophagy-dependent cell death and apoptosis	HCC‐1937 (IC_50_ = 0.25 μM) MDA-MB-231 (IC_50_ = 0.5 μM) BT-579 (IC_50_ = 2 μM)	BL1 M MSL	[305]
	Compound 7C (3-bromo-N'-(4-hydroxybenzylidene)-4-methylbenzohydrazide derivatives)	mTOR inhibitor	Active autophagy-dependent cell death and apoptosis	MDA-MB-231 (IC_50_ = 3.38 ± 1.01 μM) MDA-MB-468 (IC_50_ = 4.30 ± 1.82 μM)	BL1 MSL	[306]
	Cantharidin	Beclin1 inhibitor	Inhibit autophagy-dependent cell death and active apoptosis	MDA-MB-231 (IC_50_ = 10 µg/ml) MDA-MB-468 (IC_50_ = 10 µg/ml)	BL1 MSL	[307]
Eupalinolide G Eupalinolide I Eupalinolide J	F1012-2	Active Akt and p38 pathway	Inhibit autophagy-dependent cell death and active apoptosis	MDA-MB-231 (IC_50_ = 6.76 ± 0.42 µg/ml) MDA-MB-468 (IC_50_ = 6.23 ± 0.32 µg/ml)		[308]
	Flubendazole	EVA1A activator	Active autophagy-dependent cell death and apoptosis	MDA-MB-231 (IC_50_ = 0.623 μM) MDA-MB-468 (IC_50_ = 0.728 μM)	BLC1 MSL	[310]
	CSC-3436	AMPK/mTOR pathway ↓	Active autophagy-dependent cell death and apoptosis	MDA-MB-231 (IC_50_ = 205 ± 3.21 nm for 48 h, IC_50_ = 148 ± 2.31 nm for 72 h)	MSL	[313]
	Jatamanvaltrate P	Cleavage of PARP and caspases ↑; Cell cycle-related Cyclin B1, Cyclin D1 and Cdc-2 ↓	Active autophagy-dependent cell death and apoptosis	MDA-MB-231 (IC_50_ = 4.32 ± 1.34 µM) MDA-MB-468 (IC_50_ = 7.05 ± 2.51 µM) MDA-MB-453 (IC_50_ = 4.05 ± 0.18 µM)	BL1 MSL LAR	[314]
	1,4,5-Oxathiazinane-4,4-dioxide (OTD)		Active necroptosis and apoptosis	BT-20 (IC_50_ = 500 μM) MDA-MB-231 (IC_50_ = 200 μM)	MSL UNS	[315]
	AEZS 126	PI3K/AKT inhibitor	Active necroptosis and apoptosis	HCC1937 (IC_50_ = 3.2 μM)	BL1	[318]
	Azobenzene combretastatin A4 (Azo-CA4)	Microtubule inhibitor	Active ferroptosis and apoptosis	MDA-MB-231 (IC_50_ = 1.02 mg/mL)	MSL	[331]
	DMOCPTL	GPX4 inhibitor	Active ferroptosis and apoptosis	MDA-MB-231 (IC_50_ = 0.34 ± 0.13 μM)	MSL	[320]

### Targeting mitotic catastrophe

Mitotic catastrophe is a type of cell death during mitosis due to abnormal chromosome separation, such as mitotic checkpoint defects and damage [193, 194]. The treatment of cancer with anticancer drugs such as DNA damage drugs and microtubule targeting could induce mitotic catastrophe [195], while inhibition of mitotic catastrophe would promote the development of chemoresistance and tumor occurrence [196–198]. PTX, as a microtubule inhibitor, interfered with microtubule dynamics by stabilizing microtubule polymerization, resulting in abnormal chromosome condensation, and finally formed mitotic catastrophe [199]. Due to the poor water solubility of PTX, currently commonly used solvents of PTX tend to result in many adverse reactions [199]. Nanodiamond (ND), as a drug delivery carbon-based nanomaterial with good biocompatibility, PTX carrying ND reduced the cell viability of TNBC and induced mitotic catastrophe in a concentration-dependent manner. Interestingly, the use of ND alone would not induce cell death [199, 200]. As a common drug for standard chemotherapy in patients with TNBC, these findings about PTX suggested that the study of mitotic catastrophe mechanism of RCD might bring a new vision and direction for the treatment of TNBC. Unfortunately, TNBC tumors were prone to only respond to traditional chemotherapy well at the beginning [201] and often relapsed within 3 years. In recent years, in a variety of cancer mice models, bromodomain and extraterminal protein inhibitors (BETi) prevented the recruitment of bromodomain and extraterminal protein (BET) protein to chromatin and inhibit BET transcriptional activity by selectively targeting the epigenetic reader of BET family [202–204]. In a variety of TNBC models, the classic BETi JQ1 could induce polynuclear, which was a characteristic early index of mitotic catastrophes [193], and followed by apoptosis and aging, leading to mitotic catastrophe [205]. Inhibition of anti-apoptotic protein Bcl-xL could promote mitotic catastrophe and apoptosis induced by BETi [206]. High Bcl-xL level may be a potential effective biomarker for BETi in the treatment of TNBC [207]. In addition to the above hot targets, more studies in recent years had focused on new targets for the treatment of TNBC. A study through the breast cancer dataset and Gene Ontology (GO) database found that *checkpoint kinase 1* (*chk1*) gene is significantly overexpressed in TNBC patients, and chk1 as a key target is involved in the DNA repair pathway to treat TNBC. The results show that chk1 inhibitor SB218078 can reduce the cell viability and survival rate of TNBC cells by inducing mitotic catastrophe [208]. In addition, the analysis of human clinical database TCGA shows that NOTCH1 is highly expressed in TNBC. The results show that NOTCH1 restores S/G2 and G2/M cell cycle checkpoints by activating rad3-related protein (ATR)-chk1 signaling pathway [209], so as to inhibit mitotic catastrophes caused by BRCA1 deficiency [210, 211]. Interestingly, TNBC had a unique cell cycle progression mechanism due to its inherent genetic complexity and was sensitive to drugs leading to mitotic catastrophes [212, 213]. Polo-like kinase 1 (Plk1)-interacting checkpoint helicase (PICH), the binding substrate of plk1 which was a key enzyme in M-phase progression, was significantly overexpressed in TNBC through clinical sample analysis, ensuring reliable chromosome separation and promoting the growth of TNBC cells. The results further showed that the depletion of PICH in TNBC cells would lead to the formation of chromatin bridge and late chromosome lag, the formation of binuclear, and finally resulted in mitotic catastrophe and apoptosis. The adenosine triphosphatase (ATPase) activity of PICH is necessary for the proliferation and survival of TNBC cells, suggesting that PICH is a potential new therapeutic target of TNBC [212]. In addition to the above single target inhibition treatment of TNBC, small-molecule drugs that exert a higher level of therapeutic effect by inhibiting a variety of targets are becoming more and more popular. Torin2 and its chemical analogues lead to the accumulation and replication disaster of single-stranded DNA by simultaneously inhibiting mTOR and other PI3K-like kinases (PIKKs), which eventually leads to mitotic catastrophes and the death of TNBC tumor cells [214]. By further developing the combination of existing mTOR and PIKKs inhibitors or torin2 analogues, it may become a potentially effective strategy for the treatment of TNBC. KX-01 downregulated the expression of phosphorylated SRC and proliferation signal molecules by inhibiting SRC and tubulin at the same time, induced G2/M cell cycle arrest, increased aneuploid cell population and multinucleated cells, and finally induced mitotic catastrophe, which effectively delayed the tumor growth of TNBC mouse xenotransplantation model [215]. The inhibition of tubulin overcame the therapeutic limitation that the current SRC inhibitors failed to show clinical benefits in the treatment of TNBC (Table 3) (Fig. 3a) [216, 217].

### Targeting necroptosis

Necroptosis is a kind of RCD, resembling necrosis-dying cells cluster, releasing intracellular components and recruiting a large quantity of inflammasomes [8]. Necroptosis shows the morphological type of necrosis. Its function in cancer was mainly to mediate the adaptive function when the stress response failed. Necrosis was mediated by three major kinases: receptor-interacting protein (RIPK) 1, RIPK3 and mixed lineage kinase domain-like (MLKL) [8]. Sometimes, TNBC had no obvious response to chemotherapy [218]. It was urgent to explore TNBC-specific signal pathway and sensitive biomarkers [219]. Aquaporin1 (AQP1), a water transport membrane protein related to tumor development and progression [220, 221], as a carcinogenic biomarker of a variety of cancers [222–224], could inhibit RIPK1/MLKL/RIPK3-mediated necroptosis and RIPK1/caspase-8/caspase-3-mediated apoptosis by binding to d324 site of ripk1, driving the progression of TNBC [225]. As a negative mediator, the ectopic expression of RIPK1 could significantly weaken the signal transduction of AQP1 [225], suggesting that RIPK1 could be used as an effective potential target for the treatment of TNBC by affecting necroptosis (Table 3) (Fig. 3b).

### Targeting ferroptosis

Ferroptosis is a subroutine of RCD caused by oxidative disturbance of intracellular microenvironment, which was related to the accumulation of toxic lipid peroxides [8]. With acquired resistance of cancer cells, ferroptosis-inducing therapy shifted its importance in recent years [226]. The escape of cancer cells from other types of RCD might be still sensitive to ferroptosis [227]. TNBC cells were sensitive to ferroptosis inducers [228, 229]. Glutathione peroxidase 4 (GPX4) [230], as the main inhibitor of ferroptosis [231], is antioxidant defense enzyme. Its deletion can specifically activate ferroptosis [232]. Gefitinib is a classical epidermal growth factor receptor (EGFR) tyrosine kinase inhibitor for the treatment of TNBC, and some TNBC subtypes are resistant to it [233, 234]. A recent study found that the expression of GPX4 increased in gefitinib-resistant cells. By constructing gefitinib-resistant TNBC cells, it was found that silencing the expression of GPX4 increased the production of MDA and ROS, reduced the level of glutathione (GSH) and finally promoted ferroptosis, resulting in the inhibition of TNBC cell viability and colony forming ability, and the improvement in sensitivity of TNBC cells to gefitinb [235]. TNBC cells are very sensitive to iron poisoning induced by erastin [236], a low molecular weight chemotherapeutic drug. Unfortunately, its application is hindered by the nephrotoxicity caused by side effects and limited water solubility. In recent years, the more advanced development of nanomaterials has made a lot of contributions to the drug delivery system of small-molecule targeted drugs [237–240]. Exosomes, as micro-membrane vesicles, have attracted extensive attention because they can be used as drug delivery carriers to load low molecular weight chemotherapeutic drugs in cancer [241–246]. Researchers had developed a formula for exosomes loaded with erastin which labeled with folate (FA) (erastin@FA-exo) [247]. By inhibiting the GPX4 expression and upregulating the dioxygenase (CDO1) expression, erastin@FA-exo could reduce the level of GSH, increase the level of ROS and promote ferroptosis in TNBC cells with FA receptor overexpression. Compared with free erastin, erastin@FA-exo strongly inhibited the proliferation and migration of TNBC cells [248]. It is suggested that exosome-based drug delivery system may provide a new choice and direction for the treatment of TNBC. Ferroptosis is often accompanied by the accumulation of lethal ROS [249]. As a pleiotropic protein, lactoferrin (Lf) was often used to study the efficacy in cancer recently [250]. As an iron-saturated Lf, hollo lactate (Holo-Lf) induced ferroptosis in TNBC tumors by increasing total iron content and promoting the production of ROS, which showed better anticancer properties than low iron-saturated Lf (Apo-Lf) (Table 3) (Fig. 3c) [251].

### Targeting pyroptosis

Pyroptosis, also known as inflammatory cell necrosis, is a subroutine of RCD mediated by gasdermin (GSDM), which is characterized by the pore formation in the plasma membrane, cell swelling and rupture of the membrane, resulting in the release of cell contents and then activating a strong inflammatory response [252–255]. On the one hand, chemotherapeutic drugs could promote the cleavage of gasdermin E (GSDME) by activating Caspase-3, to transform apoptosis into pyroptosis and promote tumor cell death [256, 257]. Likewise, tetraarsenic hexoxide (As_4_O_6_) could inhibit the phosphorylation of mitochondrial STAT3 and activate mitochondrial ROS-mediated GSDME pathway, to induce pyroptotic cell death in TNBC cells, and finally inhibit tumor growth and metastasis of TNBC [258, 259]. On the other hand, chemotherapeutic drugs also played an anti-TNBC role by inducing pyroptosis [260]. Analogously, cisplatin is a classic chemotherapy drug for main mechanism of actions (MOA), is DNA damage, and induced pyroptosis through non-major (or off-target) drug effects by upregulating the long noncoding RNA (lncRNA) *maternally expressed gene 3* (*MEG3*) and activating the NLR family, pyrin domain containing 3 (NLRP3)/caspase-1/gasdermin D (GSDMD) pathway, to treat TNBC [260, 261]. Interestingly, polyI: C, a synthetic double-stranded RNA (dsRNA) analogue traditionally used to activate retinoic acid-inducible gene-I (RIG-I)-like receptors (RLRs), promoted tumor cell death by inhibiting the anti-pyroptotic function of TGF after transfection into TNBC cells (Table 3) (Fig. 4a) [262].

### Targeting anoikis

Anoikis is a special form of programmed cell death induced by the loss of contact between cells and extracellular matrix (ECM) and other cells (Table 3) (Fig. 4b) [8]. The growth of most cancer cells depends on anchoring. Without attachment to ECM in vivo, cancer cells would experience anoikis [263, 264], which played an important role in tumor metastasis [265]. Activating anoikis was the key factor to resist the occurrence and development of tumor. Protein kinase c theta (PRKCQ)/protein kinase C theta (PKCθ), as a member of the novel protein kinase C (PKC) family [266], was a regulatory factor that does not depend on adherence to survival in breast cancer cells [267], and was preferentially expressed in TNBC [268]. The results proved that PRKCQ/PKCθ promoted the phosphorylation of retinoblastoma (Rb), caused growth factor-independent cell cycle arrest, promoted the formation of tumor phenotypes [269, 270] and enhanced anchorage-independent survival, proliferation and migration [268]. Downregulation of the expression level of PRKCQ/PKCθ promoted anoikis of TNBC cells and inhibited the growth of TNBC xenografts [268]. The results showed that AEB071 as a PKCθ inhibitor also inhibited the growth of TNBC cells [268]. Research on PRKCQ/PKCθ promoting the growth of TNBC in vitro and in vivo supported its use as a potential and effective target in the treatment of TNBC. Moreover, a study manifested that synthesized flavor-derived GL-V9 could reduce glucose-6-phosphate dehydrogenase (G6PD) and increase phospho-acetyl-CoA carboxylase (p-ACC) by activating the activity and expression level of AMPK, and finally activate anoikis to inhibit the tumor metastasis of MDA-MB-231 TNBC cell line and TNBC xenograft nude mice [271]. Tubeimoside V (TBMS-V) activated EGFR and ITGB1-FAK by regulating caveolin-1 (cav-1)-related signal pathway [272] and finally made TNBC cancer cells sensitive to anoikis and inhibited TNBC cell growth and metastasis [273]. Disulfiram/copper (DSF/Cu) induced anoikis and significantly inhibited TNBC cell migration and invasion by activating calpain and decomposing vimentin in a Cu-dependent manner [274]. In TNBC xenograft tumor model, DSF also inhibited lung nodule growth and tumor growth by activating calpain [274]. Unfortunately, not all cancer cells could be affected by anoikis. Gaining resistance to anoikis had been identified as a feature of the treatment of advanced cancer cells and a key step in the process of tumor metastasis [275, 276]. CD44^+^/CD24^−^ stem cell-like population in TNBC tended to be a more aggressive phenotype [277–281], and cancer stem cell (CSC) was resistant to anoikis by allowing replication independent of anchoring [282, 283]. STAT3 could regulate stem cell self-renewal and differentiation and resist anoikis [284, 285]. It was also overexpressed and structurally activated in TNBC cells. During anchoring independent growth, salinomycin could reduce CD44^+^/CD24^−^ stem cell-like population and inhibit the formation of mammary gland ball. In the meantime, salinomycin exerted a significant inhibitory effect on TNBC cell migration and invasion. Mechanically, salinomycin could not only downregulate MMP-9 and MMP-2 messenger ribonucleic acid (mRNA) levels, but also activate caspase-3 and caspase-8, cleave PARP and inhibit STAT3 phosphorylation tyr705, finally significantly inducing anoikis sensitivity [286]. CSC can also overexpress the chemokine receptor C-X-C motif chemokine receptor 4 (CXCR4) in many cancer types by using the typical pathway of hematopoietic stem cells (HSCs) [287–293]. POL5551, as a peptidic CXCR4 antagonist, enhanced the susceptibility of tumor cells to anoikis by mobilizing tumor cells into surrounding blood and significantly reduced distant metastasis in TNBC in situ model [294]. The interaction between chemokine and its homologous receptors is very important in tumor metastasis. In addition to the above studies on chemokine receptor CXCR4, chemokine receptor C–C motif chemokine receptor 7 (CCR7) was also found to be a sialylated protein and highly expressed in human breast cancer cells [295]. The homologous ligand chemokine (C–C motif) ligand 19 (CCL19) of CCR7 prevented anoikis and increased invasion by upregulating the survival promoting proteins Bcl-2 and Bcl-xL [296]. Sialyltransferase inhibitor AL10 restrained the proliferation and invasion of TNBC cells by suppressing the abnormal sialylation of CCR7 and then triggering anoikis [296]. Some studies had established a more aggressive anti-anoikis TNBC cell. BBR could promote the growth inhibition of anoikis-resistant TNBC cells by inducing cell cycle arrest at G0/G1 phase, which was more effective than traditional Adriamycin treatment for breast cancer [276]. In addition, HPW-RX40 restored the anoikis sensitivity of TNBC cells resistant to anoikis and induced cell death by reducing the activation and expression of β1 integrin and inhibiting the FAK pathway [297]. Interestingly, in addition to regulating the classic anoikis pathway as described above, certain small-molecule drugs could induce anti-anoikis mechanisms [298]. For instance, vacuolar ATPase (V-ATPase) is a proton pump located on the membrane of acidic organelles, which affected anoikis by regulating receptor recirculation through acidification of endosomes and lysosomes [299–301]. A V-ATPase inhibitor archazolid A induced reactive oxygen species and resists anoikis by promoting late BIM degradation mediated by ERK, Src and Akt kinase [298]. The results showed that archazolid A treatment inhibited the metastasis of TNBC cancer cells and reduced the lung metastasis of mouse breast cancer cells in vitro [298]. Inhibition of V-ATPase provides us with a unique perspective to inhibit TNBC cancer metastasis and study anoikis resistance [302].

## Combination therapies of RCD subroutines with small-molecule compounds in TNBC

### The interrelationships between different RCD subroutines

Autophagy and apoptosis are the main types of RCD of eukaryotic cells. Many strategies for treating TNBC concentrated on regulating apoptosis and autophagy to inhibit cancer initiation and development. Interestingly, autophagy, as a double-edged sword of cancer cells, achieved the purpose of treating cancer by promoting autophagy or inhibiting autophagy in different tumor microenvironments [303]. The induction of cytoprotective autophagy could promote the survival of TNBC cells [304]. Narciclasine could promote autophagy-dependent apoptosis in a dose-dependent manner by upregulating AMPK-ULK1 signal axis [305]. The latest study adopted the structure simplification strategy and obtained N-(1H-benzo[d].imidazole-2-yl)-4-(1-(2-(3-bromobenzoyl)hydrazono)ethyl)benzamide (compound 7C) with excellent mTOR enzyme inhibitory activity through virtual screening and bioactivity determination based on pharmacophore [306]. Compound 7C could induce autophagy cell death and apoptosis in TNBC cell line and exhibited the most effective inhibitory activity on TNBC cells among the analogous synthetics [306]. Interestingly, the inhibition of autophagy flux could also treat TNBC. Cantharidin, a terpenoid compound, could inhibit the transformation from LC3 I to LC3 II and the formation of autophagy with a significant inhibitory activity to cancer in TNBC cells and TNBC nude mouse models. Mechanically, Cantharidin suppressed the expression of beclin-1, finally inhibiting autophagy and inducing apoptosis [307]. Similarly, a new SL active component F1012-2, which consisted of three compounds, namely Eupalinolide G, Eupalinolide I and Eupalinolide J, isolated from Eupatorium lindleyanum DC could induce apoptosis in a caspase-dependent manner through endogenous and exogenous pathways, and the induced apoptosis could be enhanced by inhibiting autophagy and finally suppressing the growth of TNBC cells [308]. Clinical trials for TNBC had a high failure rate, and targeted therapeutic drugs for TNBC were rarely approved by Food and Drug Administration (FDA). Therefore, search for novel approaches of approved drugs might be a very promising and potential strategy for the treatment of TNBC [309]. Flubendazole, a broad-spectrum antibody drug, had been repositioned as a promising anticancer drug. Flubendazole regulated autophagy and apoptosis by targeting the key site thr113 of EVA1A and finally inhibited the proliferation and migration of TNBC [310].

Autophagy can usually prolong the survival time of cancer cells by removing damaged organelles and providing nutrients for cancer cells. Autophagy can also maintain the homeostasis of genome and internal environment, prevent inflammation or oxidative stress, and inhibit the occurrence, proliferation and metastasis of tumor cells [311, 312]. A series of small-molecule compounds targeting autophagy-related proteins (or autophagy process) have shown good anticancer effects in cancer cells. Therefore, targeted autophagy has great potential for the treatment of TNBC patients. However, the relationship between autophagy and apoptosis is not clear. The rationale for using autophagy inhibitors in combination with chemotherapeutic drugs is a better way to improve the efficacy of anticancer treatments and counteract TNBC resistance. Tamoxifen is a selective estrogen receptor modulator (SERMs). Due to the negative expression of ER receptor in TNBC, tamoxifen has a poor prognosis and even drug resistance against TNBC. A study found that tamoxifen induces autophagy in TNBC. Combined treatment with csc-3436 enhanced the tumor growth inhibitory effect of tamoxifen on TNBC compared with tamoxifen alone in vivo. The molecular mechanism may be that CSC-3436 converts tamoxifen-induced autophagy into apoptosis through AMPK/mTOR pathway and cleavage of ATG-5 [313]. Jatamanvaltrate P enhances the cleavage of PARP and caspase, while reducing the expression levels of cell cycle-related cyclin B1, cyclin D1 and cdc-2. It plays its cytotoxic and antitumor role in TNBC cell lines (MDA-MB-231, MDA-MB-453 and MDA-MB-468) and MDA-MB-231 xenografts by inducing apoptosis, and autophagy-dependent cell death [314].

Recently, a new antitumor drug 1,4,5-oxathiazinane-4,4-dioxide (OTD) was designed and synthesized [315]. The results showed that OTD induced necroptosis and apoptosis of TNBC cells, resulting in cell death and inhibition of proliferation in the dose-dependent manner [315]. Clinical studies showed that the poor prognosis of TNBC was related to the activation of PI3K/AKT pathway [316, 317]. A recent study exhibited that PI3K/AKT inhibitor AEZS 126 caused cell death by inducing apoptosis and necroptosis in TNBC cells [318]. Among the basal-like subtypes of TNBC, chemotherapy combined AEZS 126 with good toxicity characteristics and antitumor activity might be a potential strategy for TNBC clinical trials. GPX4 is an important ferroptotic cell death regulator [319], and its expression in TNBC is higher than that in other subtypes of breast cancer [320]. The decrease in GPX4 expression can induce apoptosis [321]. As a derivative of natural product parthenolide, DMOCPTL significantly inhibited the proliferation of TNBC cells by directly binding to GPX4 protein and induced GPX4 ubiquitination to promote ferroptosis. Also, DMOCPTL could upregulate EGR1, resulting in the activation of mitochondrial-mediated apoptosis [320]. The metabolic characteristics of cancer cells are at least partly attributed to the imbalance of specific amino acids and abnormal metabolism of amino acids [322–324]. A present study found that TNBC was highly sensitive to cystine starvation. Cystine starvation in TNBC cells increased the phosphorylation at eIF2α serine 51 and the protein expression levels of ATF4 and CHAC1 by activating GCN2, induced mitochondrial rupture, dysfunction, and ROS production, reduced the level of GSH, and induced necroptosis and ferroptosis, resulting in cell death [236, 322, 325].

Chemotherapy is a common scheme for the treatment of TNBC; however, the non-specific distribution and non-targeted side effects of chemotherapy limit its long-term application for patients [326]. The increasing development of nanomedicine was expected to solve these issues by specifically activating drug efficacy [327–330]. A photo-switchable microtubule inhibitor azobenzene combretastatin A4 (Azo-CA4) was loaded into up-conversion nanocarriers to promote microtubule decomposition and cell cycle arrest in G2/M phase by inducing Azo-CA4 photoisomerization, resulting in cell apoptosis. The reduction of Fe^3+^ to Fe^2+^ induced by ultraviolet light caused ferroptosis, significantly reduced the activity of TNBC cells and inhibited the tumor size of xenograft mouse model [331]. Although chemotherapy is widely used during TNBC treatment, the side effect was extremely serious according to the evaluation based on Common Terminology Criteria for Adverse Events. Moreover, chemotherapy could threaten the health of patients with high dosage. Even combining chemotherapy with radiation could not reduce the risk of recurrence and metastasis, or abate the threat of adverse effects [332]. In recent years, an increasing number of researchers focused on unconventional methods, such as bacterial toxins. LT-IIc, a member of the bacterial type II subfamily of heat-labile enterotoxin, which could promote TNBC cell death by inducing apoptosis and necroptosis [333].

### Combination therapies of small-molecule compounds

Pharmacological regulation by inducing or inhibiting autophagy [334, 335], combined application of inducing apoptosis to produce effective innovative strategies, could enhance therapeutic activity. The combined application of torin-1 (TOR) and chloroquine (CQ) could show a half-lethal effect by inducing autophagic cell death and apoptotic cell death [336]. The combined application of CQ and DOX induced AVO accumulation through non-major (or off-target) drug effects by inhibiting apoptotic cell death and ultimately increased growth inhibition [336]. Copper (I) nicotinate complex (CNC) combined with DOX could inhibit autophagy by inducing cell cycle arrest and reduced the dose of DOX applied to TNBC cells [336]. The expression level of EGFR was higher in 40% of TNBC patients [337, 338]. However, EGFR inhibitors are mostly ineffective in the treatment of TNBC patients, and increasing evidence supports that autophagy is currently related to the drug resistance of EGFR inhibitors in the treatment of TNBC. Inhibition of autophagy by 3-methyladenine or bafilomycin A1 increased the sensitivity of gefitinib to TNBC cell lines and more strongly inhibited cell viability and colony formation by inducing G0/G1 arrest and DNA damage and activating mitochondrial-dependent apoptosis pathway. The combined application of autophagy inhibitor and gefitinib also improved the antitumor effect in TNBC nude mouse model. These results suggest that targeted autophagy should be considered as an effective therapeutic strategy to enhance the sensitivity of EGFR inhibitors to TNBC [339]. ND, a carbon-based nanomaterial, combined PTX and cetuximab (CET) for the targeted treatment of EGFR-positive TNBC cells. The results showed that ND-PTX-CET enhanced the mitotic catastrophe and apoptosis of TNBC cells by targeting EGFR, which provided a feasible strategy for the treatment of TNBC based on RCD synergistic mechanism [200]. Cetuximab was a classic monoclonal antibody against EGFR [340–343]. However, with the advancement of the course of treatment, cancer cells rapidly gained resistance to cetuximab. Hence, it is urgent to develop novel combination therapy [344]. MicroRNA (miRNA)-155-5p was found to be upregulated in TNBC cells as a new biomarker [345]. By upregulating gasdermin E-N-terminal subunit (GSDME-N) and cleaving caspase-1 [346], miR-155-5p antagonist combined with cetuximab could promote apoptosis and pyroptosis and inhibit the proliferation and migration of EGFR overexpressing TNBC cells. Meanwhile, miR-155-5p antagonist also enhanced the antitumor effect of cetuximab in TNBC xenotransplantation model [347]. Cancer cells with anoikis resistance are prone to metastasis [348], which is reported to be related to integrin and growth factor receptor [349–354]. HPW-RX40, which inhibited integrin, combined with EGFR inhibitor AG1478, could inhibit FAK/paxillin phosphorylation and significantly induce TNBC cell death [297]. Blocking both integrin and growth factor-dependent survival pathways may be a potential strategy to overcome the resistance of TNBC cancer cells to EGFR inhibitors [355]. Chk1 inhibitor could develop as a single drug [208]; in order to improve its anti-TNBC tumor effect, it is worthwhile to explore the potential synergistic effect of chk1 inhibitors and chemotherapy. A recent study found that the long-term treatment of DNA damaging agent carboplatin, as a cycle nonspecific anticancer drug for main mechanism of actions (MOA), is DNA damage and inhibits tumor growth and could induce the mitotic arrest through non-major (or off-target) drug effects of TNBC. The combined application of carboplatin and chk1 inhibitor AZD7762 inhibited the G2-M conversion by inhibiting chk1 pathway, resulting in the accumulation of DNA damage, a significant increase in the incidence of spindle multipolarity and cytokinesis failure. The co-treatment promoted the polynuclear and polyploidization of TNBC tumor cells, eventually leading to mitotic catastrophe and apoptosis. The results showed that the combination of DNA damaging agent and chk1 inhibitor could synergistically inhibit the growth and proliferation of multiple TNBC cell lines in vitro [356, 357]. In addition, ganetespib could also promote mitotic catastrophe and apoptosis when used in combination with docetaxel in vitro [358]. Similarly, their combination exerted significantly synergistic activity in vivo [358]. It is revealed that the synergistic induction of mitotic catastrophe and apoptosis can be used as an alternative strategy for the treatment of TNBC. Interestingly, recent studies achieved effective results in the treatment of TNBC through combination therapy alone or in parallel with chemotherapy. ATR inhibitor AZD6738 and WEE1 inhibitor AZD1775 inactivated RAD51-mediated homologous recombination [359], activated cyclin-dependent kinase 1 (CDK1) activity, forced DNA-damaged cells into mitosis, and induced serious mitotic abnormalities and mitotic catastrophes, eventually resulting in TNBC cell death, and improving the sensitivity of TNBC to cisplatin and PARP inhibitors [360, 361]. BMS-754807, as a dual IGF-1R/InsR inhibitor, combined with the chemotherapeutic drug docetaxel could increase cell apoptosis and induce mitotic catastrophe, inhibit the growth of TNBC primary human tumor transplantation MC1, and regress tumors [362]. Additionally, the combination of IGF-1R inhibitor NVP-AEW541 and autophagy inhibitor 3-mA could improve the therapeutic effect of IGF-1R inhibitors in TNBC cells, which provided a direction for the combined treatment strategy based on IGF-1R inhibitors [363]. Multiple schemes in parallel revealed an innovative and effective targeted therapy in TNBC. Functional studies, clinical dataset analysis and breast cancer specimens showed that TNBC had unique vulnerability to ferroptosis inducers [364, 365], and BRD4 transcripts as well as protein levels were significantly enriched in TNBC [366–368]. The results showed that classical bet inhibitor JQ1 and proteasome inhibitor bortezomib (BTZ) showed effective synthetic lethality to major TNBC subtypes by inducing ferroptosis [369]. Likewise, BET inhibitor JQ1 and CXCR2 inhibitor SB225002 had high efficacy in CO-inhibition of M/MSL TNBC subtypes by inducing apoptosis in vivo and in vitro [369]. These effective combination therapies revealed the inherent susceptibility of TNBC to ferroptosis and highlighted the potential strategy of ferroptosis as a drug target of TNBC. The expression level of mucin 1 (MUC1) transmembrane glycoprotein increased in most TNBC [370, 371]. In this case, erastin is ineffective in inducing ferroptosis [372]. Application of xCT inhibitor sulfasalazine could inhibit *MUC1* gene transcription by enhancing histone and DNA methylation on MUC1 promoter [372]. The amplification of *MUC1* gene led to over-expression of the MUC1 C-terminal subunit (MUC1-C) complex, which could mediate the TNBC cell self-renewal ability and tumor origin [373]. The results showed that xCT inhibitor reduced the expression level of MUC1-C by inhibiting MUC1-C/xCT pathway, induced ferroptosis and finally inhibited the survival rate of TNBC cells [372]. These findings suggested the prospect of the combination of xCT inhibitor and erastin in the treatment of TNBC.

In addition to the conventional combination therapy, virotherapy destroys malignant tumors without damaging normal tissues, making oncolytic virus a promising antitumor drug. A recent study found that Dox could specifically enhance the oncolytic effect of M1 virus, promote virus replication in tumor, further trigger apoptosis and necroptosis by non-major (or off-target) drug effects and significantly inhibit the tumor growth of TNBC in vivo [374]. These data indicated the combination of molecular diagnosis and viral therapy as a promising approach for the development of anti-TNBC strategies. In addition, the combination of radiotherapy and ferroptosis induced exerted coefficient effect on TNBC by affecting ROS [375]. Ferroptosis could make cancer cells more sensitive to radiotherapy [376]. Recently, Holo-Lf was found to promote ROS generation and improved the hypoxic microenvironment by reducing the expression of HIF-1α in TNBC cells. Concurrently, Holo-Lf induced ferroptosis and ultimately promoted radiation-induced DNA damage [375]. The combined application of Holo-Lf and radiotherapy enhanced the sensitivity of TNBC to radiotherapy (Table 4).

**Table 4:** Combination therapies of RCD-targeted small-molecule compounds in TNBC

Compound 1 (Name in the literature and chemical structure)	Compound 2 (Name in the literature and chemical structure)	Coordination mechanism	TNBC subtype	References
Torin-1 (mTOR inhibitor)	Chloroquine (TLRs inhibitor)	Active autophagy-dependent cell death and apoptosis	BL2	[336]
Chloroquine (TLRs inhibitor)	Doxorubicin (Topoisomerase l/II inhibitor)	Inhibit apoptosis	BL2	[336]
CuCl(HNA)_2_ Copper (I) nicotinate complex (CNC)	Doxorubicin (Topoisomerase l/II inhibitor)	Inhibit autophagy-dependent cell death	BL2	[336]
Gefitinib (EGFR inhibitor)	3-MA (Autophagy-dependent cell death inhibitor)	Inhibit autophagy-dependent cell death and active apoptosis	BL1 MSL	[339]
PTX (Microtubule inhibitor)	Cetuximab	Active mitotic catastrophe and apoptosis	MSL	[200]
HPW-RX40 (Integrin inhibitor)	AG1478	Active anoikis	MSL	[297]
AZD7762 (chk1 inhibitor)	Carboplatin (DNA-damaging agents)	Active mitotic catastrophe and apoptosis	BL1 MSL	[356]
AZD6738 (ATR inhibitor)	AZD1775 (WEE1 inhibitor)	Active mitotic catastrophe	MSL	[359]
Ganetespib (HSP90 inhibitor)	Docetaxel	Active mitotic catastrophe and apoptosis	UNS	[358]
BMS-754807 (IGF IR/InsR inhibitor)	Docetaxel	Active mitotic catastrophe and apoptosis		[362]
NVP-AEW541 (IGF IR inhibitor)	3-MA (Autophagy-dependent cell death inhibitor)	Inhibit autophagy-dependent cell death	M MSL	[363]
JQ1 (BET inhibitor)	Bortezomib (proteasome inhibitor)	Active ferroptosis	BL1 BL2	[369]
JQ1 (BET inhibitor)	SB225002 CXCR2 inhibitor	Active apoptosis	M MSL	[369]
Erastin (ferroptosis inducer)	Sulfasalazine (xCT inhibitor)	Active ferroptosis	BL1 UNS	[372]

## Targeted small-molecule compounds in TNBC clinical trials

In preclinical and clinical trials, a series of small-molecule compounds are used to explore their therapeutic effects against TNBC, and some of them have displayed remarkable results. For example, as a dual inhibitor of PI3K and HDAC, CUDC-907 has shown significant anticancer effects on TNBC cell lines in preclinical studies. When it combined with TRAIL, the cleavage of caspase-8, caspase-9 and PARP increased and induced breast cancer cell apoptosis. Besides, CUDC-907 could upregulate the expression of DR5 and downregulate the level of XIAP, Bcl-2 and Bcl-xl to promote apoptosis mediated by TRAIL. A phase 1 clinical trial (NCT02307240) evaluated the safety, tolerability and validity of CUDC-907 in advanced/relapsed solid tumors, including TNBC, high-grade serous ovarian cancer (HGSOC) and NUT midline carcinoma (NMC) [377]. In addition, ONC201, also as a TRAIL-inducing compound, could trigger apoptosis by targeting DR5. In a phase 2 clinical trial (NCT03733119), Akt/ERK inhibitor ONC201 and a methionine-restricted (MR) diet were studied in the treatment of metastatic TNBC. ONC201 could target tumor cells and cleared them without affecting normal cells, and MR diet could enhance the activity of ONC201. In another phase 2 clinical trial (NCT03394027), ONC201 was proved to kill breast cancer and endometrial cancer cells, but was not sure if ONC201 help shrink tumors of TNBC or endometrial cancers [378]. Etoposide (ET) could stimulate apoptosis with TRAIL and when ET, in combination with Dox, could markedly upregulate DR5 expression by non-major (or off-target) drug effects in TNBC cells. A phase 2 clinical trial (NCT04452370) evaluated the effect of the oral topoisomerase-II inhibitor etoposide combined with the anlotinib in the treatment of recurrent or metastatic TNBC [379].

Additionally, ENMD-2076 was a small-molecule inhibitor that was cytotoxic to p53-mutated TNBC cell lines. Significant antitumor, antiproliferative and proapoptotic activities of ENMD-2076 were observed in TNBC cells. The increased expression of p53 and p73 protein could enhance the sensitivity of cancer cells to treatment. A phase 2 clinical trial (NCT01639248) showed that ENMD-2076 could lead to partial response or clinical benefit lasting more than 6 months in patients with previously treated locally advanced or metastatic TNBC [380, 381]. NVP-BEZ235 as a PI3K/mTOR inhibitor was found to induce autophagy by degrading mutant p53 protein. Meanwhile, autophagy promoted by BEZ235 was also related to the downregulation of Akt/mTOR pathway. NVP-BEZ235, the combination of MEK1/2 inhibitor and MEK162 in phase 2 clinical trial (NCT01337765), evaluated the safety and preliminary antitumor activity in the treatment of TNBC, pancreatic cancer and other advanced solid tumors [382]. It was worth noting that more and more preclinical and clinical activities had verified the promising strategy of small molecular compounds to intervene TNBC by activating mitotic catastrophe. Clinical evidence of tumor regression and preclinical activity profile (NCT01677455) showed that ganetespib, as a selective HSP90 inhibitor, could inhibit cell growth in TNBC cell line and inhibit lung metastasis in xenotransplantation model by enhancing DNA damage and mitotic catastrophe and inactivating a variety of carcinogenic pathways. Metastatic lung and lymphatic lesions were also suppressed significantly in patients with ganetespib monotherapy [358] (Table 5).

**Table 5:** Small-molecule compounds targeting RCD subroutines in TNBC clinical trials

Name in the literature and chemical structure	Target	Mechanism in RCD	Biological activity	TNBC subtype	Clinical trial identifier	References
CUDC-907	DR5↑	Induce apoptosis	MDA-MB-231 (IC_50_ = 0.2 − 0.5 μM)	MSL	NCT02307240 (phase 1)	[377]
ONC201	TRAIL/DR5↑	Induce apoptosis	SUM149PT (GI_50_ = 2 μM); MDA-MB-468 (GI_50_ = 2 μM)	BL2, BL1	NCT03733119 (phase 2), NCT03394027 (phase 2)	[378]
Etoposide (ET)	DR5↑	Induce apoptosis	MDA-MB-231 (IC_50_ = 30 − 40 μM)	MSL	NCT04452370 (phase 2)	[379]
ENMD-2076	p53↑ p73↑	Induce apoptosis	MDA-MB-468, MDA-MB-231, HCC1187, Hs578T (Average: IC_50_ = 1.4 μM)	BL1, MSL, IM	NCT01639248 (phase 2)	[380, 381]
NVP-BEZ235	mutp53↓ Akt/mTOR↓	Induce autophagy-dependent cell death	MDA-MB-231 (GI_50_ = 0.02 − 0.04 μM) MDA-MB-468 (GI_50_ = 0.01 − 0.02 μM)	MSL, BL1	NCT01337765 (phase 1)	[382]
Ganetespib	HSP90 inhibitor	Active mitotic catastrophe	MDA-MB-231 (IC_50_ = 20 nM)	MSL	NCT01677455 (phase 2)	[358]

^*^↓, decrease/inhibition; ↑, increase/activation

## Concluding remarks and future perspectives

Cell death occurs in many forms to cope with different environmental challenges, and it has been gradually recognized that RCD can involve much more than the classical apoptosis pathway. Particularly, autophagy, necrosis and ferroptosis, belonging to RCD, follow their specific mechanistic procedures and appear in the corresponding conditions to decide cell fate. Abnormal cell growth without typical RCD can lead to many types of disease, including TNBC, which is known as the “hidden killer of women” due to its aggressive properties and limited treatment options. More recently, numerous factors of RCD have been found to be associated with the occurrence and progression TNBC. For instance, TNBC is one of the earliest genetic diseases found to be related to autophagy dysfunction. Autophagy dysfunction can help to inhibit the attack of T-lymphocytes on TNBC tumors, aiding immune escape for these tumors. Under some circumstances, autophagy can degrade tumor cells and inhibit tumor growth and metastasis through its own actions [383], suggesting that targeting autophagy will be an effective therapeutic strategy.

Hitherto, a promising area for small-molecule drug discovery has been taking an advantage of the concept that precisely targeting different molecular characteristics of TNBC subtypes. For instance, the LAR tumor characterized by AR expression is a subtype of TNBC, which is sensitive to the endocrine regulation of AR antagonists, such as enzalutamide [384]. The classifications of TNBC can be utilized as a prognostic or predictive means for better individualized therapies in TNBC patients theoretically. The online tool “TNBCtype-4” (also known as “Lehmann Classifier”) can be used for Lehmann typing, to make more accurate diagnosis, biomarker selection, drug discovery and more appropriate therapeutic strategies in TNBC [385, 386]. With the deepening of the biological behavior and molecular typing of TNBC, the development of more clinical studies and the optimization of different approaches, the diagnosis and treatment of TNBC will be more accurate and individualized.

Although small-molecule drugs have achieved encouraging results for TNBC patients and brought a new hope, they still face many challenges. Firstly, the efficacy of small-molecule drugs is based on the joint standard scheme. If they are not used according to the drug standards, the side effects of drugs will increase, the tolerance of patients will decrease, and ultimately the therapeutic efficacy will be poor. Secondly, small-molecule drug monotherapy can obtain an ideal TNBC tumor cell inhibition, but it is prone to drug-resistant mutations. Multidrug resistance sites are easy to appear after small-molecule drugs used. With the continuous development of such small-molecule drugs with low resistance, high curative effect and few side effects, as well as the research of new drug combination schemes, it is believed that it will bring a new development for the treatment of TNBC.

In summary, it is valuable for development of targeted therapies to conduct more in-depth investigations of RCD on the purpose of more accurately determining the intricate molecular mechanisms of each subroutine of RCD, and further exploring the relationships between them. With a better understanding of the complex regulatory mechanisms of RCD, we can anticipate a breakthrough for discovery of more targeted small-molecule drugs for fighting TNBC in the future.

## Data Availability

Not applicable.

## Abbreviations

ACD: Accidental death
AIF: Apoptosis-inducing factor
AKT: Protein kinase B
AMPK: Adenosine 5′-monophosphate-activated protein kinase
Apaf-1: Apoptotic protease activating factor 1
AQP1: Aquaporin1
As4O6: Tetraarsenic hexoxide
ASC: Apoptosis-associated speck-like protein containing a CRAD
ATF3: Activating transcription factor 3
ATG: Arctigenin
ATG: Autophagy-associated protein
ATP: Adenosine triphosphate
ATPase: Adenosine triphosphatase
ATR: Rad3-related protein
AURKA: Aurora kinase A
AXL: Anexelekto
Azo-CA4: Azobenzene combretastatin A4
BAM: Budlein A methylacrylate
BBC3: Bcl-2 binding component 3
BBR: Berberine
Bcl-2: B-cell lymphoma 2
Bcl-xL: B-cell lymphoma extra-large
BET: Bromodomain and extraterminal protein
BETi: Bromodomain and extraterminal protein inhibitors
Bim: Bcl-2 interacting mediator of cell death
BL1/2: Basal-like 1/2
BRD4: Bromodomain 4
BTZ: Bortezomib
CA: Cinnamic acid
Cav-1: Caveolin-1
CCL19: Chemokine (C–C motif) ligand 19
CCR7: C–C motif chemokine receptor 7
CET: Cetuximab
C-FLIP: Cellular FLICE-inhibitory protein
CDK: Cyclin-dependent kinase
Chk1: Checkpoint kinase 1
CHOP: C/EBP homologous protein
CNC: Copper (I) nicotinate complex
CSC: Cancer stem cell
Cu: Copper
Cur: Curcumin
CXCR4: C-X-C motif chemokine receptor 4
Cyt-c: Cytochrome c
dATP: Deoxyadenosine triphosphate
DIABLO: Direct IAP-binding protein with low pI
DISC: Death-inducing signaling complex
DNA: Deoxyribonucleic acid
Dox: Doxorubicin
DR3/4/5: Death receptor 3/4/5
DSF: Disulfiram
dsRNA: Double-stranded RNA
e.g.: Exempli gratia
EC50: Median effective concentration
ECM: Extracellular matrix
EGFR: Epidermal growth factor receptor
EMT: Epithelial–mesenchymal transition
ER: Estrogen receptor
ERK: Extracellular signal-regulated kinase
ET: Etoposide
FA: Folate
FADD: Fas associated via death domain
FDA: Food and Drug Administration
FIP200: Focal adhesion kinase interacting protein of 200 kD
FoxO: Forkhead box O
G6PD: Glucose-6-phosphate dehydrogenase
GA: Gallic acid
GO: Gene ontology
GPT: Ginsenoside panaxatriol
GPX4: Glutathione peroxidase 4
GSDM: Gasdermin
GSDMD: Gasdermin D
GSDME: Gasdermin E
GSDME-N: Gasdermin E-N-terminal subunit
GSH: Glutathione
HER2: Human epidermal growth factor 2
HGSOC: High-grade serous ovarian cancer
Holo-Lf: Hollo lactate
HSCs: Hematopoietic stem cells
HSP70: Heat shock protein 70
i.e.: Id est
IC50: Half maximal inhibitory concentration
IKB: Inhibitor of nuclear factor kappa-B
IKK: Inhibitor of nuclear factor kappa-B kinase
IKKβ: Inhibitor kappa-B kinase-β
IL-6: Interleukin-6
IM: Immunomodulatory
IRAK1: Interleukin-1 receptor-associated kinase 1
KRT15: Keratin 15
LAR: Luminal androgen receptor
LC3: Light chain 3
Lf: Lactoferrin
lncRNA: Long noncoding RNA
M: Mesenchymal
MAPK: Mitogen-activated protein kinase
mAtg13: Mammalian autophagy-associated protein 13
Mcl-1: Myeloid cell leukemia-1
MDM2: Murine double minute 2
MDSCs: Myeloid-derived suppressor cells
MEG3: Maternally expressed gene 3
MEK: Mitogen-activated protein kinase kinase
MIF: Migration inhibitory factor
miRNA: MicroRNA
MLKL: Mixed lineage kinase domain-like
MMP: Matrix metallopeptidase
MOM: Mitochondrial outer membrane
MR: Methionine-restricted
mRNA: Messenger ribonucleic acid
MSL: Mesenchymal stem-like
mTOR: Mammalian target of rapamycin
mTORC: Mammalian target of rapamycin complex
MUC1: Mucin 1
MUC1-C: MUC1 C-terminal subunit
NCCD: Nomenclature of Cell Death Committee
ND: Nanodiamond
NF-κB: Nuclear factor kappa-B
NLRP3: NLR family, pyrin domain containing 3
NMC: NUT midline carcinoma
OL: Oleuropein
OTD: 1,4,5-Oxathiazinane-4,4-dioxide
p-ACC: Phospho-acetyl-CoA carboxylase
PAD: Peptidylarginine deiminase
PARP1: Poly-ADP-ribose polymerase 1
PCB 104: Polychlorinated biphenyl 104
PDGFR-β: Platelet-derived growth factor receptor β
PD-L1: Programmed cell death ligand 1
PH: Phloretin
PI3K: Phosphatidylinositol 3 kinase
PI3KC: Phosphatidylinositol 3 kinase complex
PICH: Plk1-interacting checkpoint helicase
PIKK: PI3K-like kinase
PKC: Protein kinase C
PKCθ: Protein kinase C theta
Plk1: Polo-like kinase 1
PMAIP1: Phorbol-12-myristate-13-acetate-induced protein 1
POLD1: Polymerase (DNA) delta 1
PR: Progesterone receptor
PRKCQ: Protein kinase c theta
PTEN: Phosphatase and tensin homologue deleted on chromosome ten
PTER: Pterostilbene
Rb: Retinoblastoma
RCD: Regulated cell death
RIG-I: Retinoic acid-inducible gene-I
RIPK: Receptor-interacting protein
RLRs: Retinoic acid-inducible gene-I (RIG-I)-like receptors
ROS: Reactive oxygen species
SAPK: Stress-activated protein kinase
SG: Sophoraflavanone G
SGNI: Shuganning injection
SHP-1: Src homology region 2 domain containing phosphatase 1
SIRT6: Silent information regulator 6
SJW: St. John's wort
SJWE: SJW extract
SLC3A2: Solute carrier family 3 member 2
SLC7A11: Solute carrier family 7 member 11
SMAC: Second mitochondria-derived activator of caspase
ssDNA: Single-stranded deoxyribonucleic acid
STAT3: Signal transducer and activator of transcription 3
TBMS-V: Tubeimoside V
TNBC: Triple-negative breast cancer
TNFR: Tumor necrosis factor receptors
TOR: Torin-1
TRADD: TNFRSF1A associated via death domain
TRAF2: TNF receptor-associated factor 2
TRAIL: TNF-related apoptosis-inducing ligand
TSC1/2: Tuberous sclerosis complex 1/2
ULK1: Unc-51-like autophagy activating kinase 1
UNS: Unclassified
V-ATPase: Vacuolar-ATPase
Vps34: Vacuolar protein sorting 34
XIAP: X-linked inhibitor of apoptosis protein
XPO-1: Exportin 1
ZnPP: Zn protoporphyrin IX
